# Elimination of Chromosomal Island SpyCIM1 from *Streptococcus pyogenes* Strain SF370 Reverses the Mutator Phenotype and Alters Global Transcription

**DOI:** 10.1371/journal.pone.0145884

**Published:** 2015-12-23

**Authors:** Christina Hendrickson, Chad W. Euler, Scott V. Nguyen, Maliha Rahman, Kimberly A. McCullor, Catherine J. King, Vincent A. Fischetti, W. Michael McShan

**Affiliations:** 1 Department of Pharmaceutical Sciences, The University of Oklahoma Health Sciences Center, Oklahoma City, Oklahoma, United States of America; 2 Department of Microbiology and Immunology, The University of Oklahoma Health Sciences Center, Oklahoma City, Oklahoma, United States of America; 3 The Biology Department, The University of Central Oklahoma, Edmond, Oklahoma, United States of America; 4 Laboratory of Bacterial Pathogenesis and Immunology, The Rockefeller University, New York, New York, United States of America; 5 Department of Medical Laboratory Sciences, Belfer Research Building, Hunter College, CUNY, New York, New York, United States of America; 6 Department of Microbiology and Immunology, Weill Cornell Medical College, New York, New York, United States of America; University of Kansas Medical Center, UNITED STATES

## Abstract

*Streptococcus pyogenes* chromosomal island M1 (SpyCIM1) integrates by site-specific recombination into the 5’ end of DNA mismatch repair (MMR) gene *mutL* in strain SF370SmR, blocking transcription of it and the downstream operon genes. During exponential growth, SpyCIM1 excises from the chromosome and replicates as an episome, restoring *mutL* transcription. This process is reversed in stationary phase with SpyCIM1 re-integrating into *mutL*, returning the cells to a mutator phenotype. Here we show that elimination of SpyCIM1 relieves this mutator phenotype. The downstream MMR operon genes, multidrug efflux pump *lmrP*, Holliday junction resolution helicase *ruvA*, and DNA base excision repair glycosylase *tag*, are also restored to constitutive expression by elimination of SpyCIM1. The presence of SpyCIM1 alters global transcription patterns in SF370SmR. RNA sequencing (RNA-Seq) demonstrated that loss of SpyCIM1 in the SpyCIM1 deletion mutant, CEM1Δ4, impacted the expression of over 100 genes involved in virulence and metabolism both in early exponential phase, when the SpyCIM1 is episomal, as well as at the onset of stationary phase, when SpyCIM1 has reintegrated into *mutL*. Among these changes, the up-regulation of the genes for the antiphagocytic M protein (*emm1*), streptolysin O (*slo*), capsule operon (*hasABC*), and streptococcal pyrogenic exotoxin (*speB*), are particularly notable. The expression pattern of the MMR operon confirmed our earlier observations that these genes are transcribed in early exponential phase but silenced as stationary phase is approached. Thus, the direct role of SpyCIM1 in causing the mutator phenotype is confirmed, and further, its influence upon the biology of *S*. *pyogenes* was found to impact multiple genes in addition to the MMR operon, which is a novel function for a mobile genetic element. We suggest that such chromosomal islands are a remarkable evolutionary adaptation to promote the survival of its *S*. *pyogenes* host cell in changing environments.

## Introduction

Prophages and prophage-like elements are universal components of the genomes of *Streptococcus pyogenes* (group A streptococcus), with the published genome sequences having between two and eight examples in each strain [1–12]. These important mobile genetic elements (MGE) provide a substantial contribution of genetic material to their hosts, often representing ~10% of the total genome. Streptococcal prophages generally follow the typical genetic organization of lambdoid phages with genes organized for coordinated expression of the establishment of lysogeny or for the expression of early and late genes in the lytic cycle [13, 14]. Importantly, superantigens and other streptococcal virulence factors are components of these prophage genomes. In addition to typical prophages, other MGEs are present in the *S*. *pyogenes* genomes that include insertion sequence (IS) elements, transposons, and chromosomal islands.

Recently, we demonstrated that a prophage-like MGE in the *S*. *pyogenes* M1 genome strain SF370 acted as a genetic switch that controlled the expression of the DNA mismatch repair (MMR) gene *mutL* as well as additional downstream genes. These genes are encoded on a polycistronic mRNA along with *mutS*, and the result of this regulation caused a growth-dependent mutator phenotype [15]. This MGE, which was originally annotated in the genome as prophage SF370.4 and is now named SpyCIM1 (***S*** *treptococcus* ***py*** *ogenes* **c**hromosomal **i**sland M1), mediated expression of the MMR operon through a process of dynamic excision and re-integration from the 5’ end of the *mutL* ORF. During a state of rapid cell division, the prophage element excises from the bacterial chromosome and replicates as a circular episome, allowing the normal expression of *mutL* and the downstream genes. When the cells approach stationary phase and division slows, the episomal form re-integrates into its attachment site in *mutL*, silencing the expression of *mutL* and downstream genes. This cycle of excision and re-integration results in the cell switching between a complex mutator and normal phenotype [15]. This system is remarkable in that not only is MMR regulated by this MGE, but it also controls a multiple drug efflux pump (*lmrP*), a Holliday junction helicase subunit (*ruvA*), and a component of base excision repair (*tag*). We extended this original observation to demonstrate that the frequent carriage of related SpyCI elements in the genomes of other *S*. *pyogenes* strains also was associated with a mutator phenotype [16].

The prophage-like element SpyCIM1 differs from typical integrated streptococcal prophages in a number of characteristics. Genetic modules for lysogeny, regulation, and DNA replication are readily identified by homology to these regions in other prophages, but no capsid or packaging structural genes, lysis genes, or virulence genes seem to be present. Further, SpyCIM1 and related elements found in other *S*. *pyogenes* genomes are smaller than typical streptococcal prophages, having a range between 13 kb to 17 kb in length [15, 16]. At first glance, such an element might be classified as a defective prophage as indeed it originally was [1, 14]. However, SpyCIM1 is a member of a sizeable group of elements in Gram-positives that follow a similar genetic organization, and it is unlikely that each chromosomal island resulted from independent prophage decay in these different genera of bacteria. So, as proposed by Novick and colleagues [17, 18], these phage-like chromosomal islands probably represent a separate class of mobile genetic elements.

Our previous studies showed that strains with SpyCI had a higher mutation rate and other phenotypic changes compared to strains lacking these MGEs; however, these earlier studies were limited to comparing *S*. *pyogenes* SpyCI^+^ strains to similar but genetically distinct ones lacking a SpyCI integrated into *mutL* [16]. Thus, the association between SpyCI carriage and a mutator phenotype was inferential and not proven due to the lack of isogenic strains for this MGE. In this report we demonstrate that the removal of SpyCIM1 from strain SF370SmR relieves the mutator phenotype by decreasing the mutation rate as well as increasing resistance to ethidium bromide, UV irradiation and ethyl methanesulfonate, which result from the restoration of constitutive expression of *mutL*, *lmrP*, *ruvA*, and *tag*. Further, loss of SpyCIM1 also altered global gene expression patterns, which were unique to growth phases of the cell. These studies confirm the impact this phage-like chromosomal island has upon its host and will be the springboard for future studies about this remarkable genetic system.

## Materials and Methods

### Strains and bacterial growth conditions

Bacterial strains and plasmids used in these studies are described in Table 1. *Escherichia coli*, used for vector propagation, was grown in Luria-Bertani (LB) broth (Difco) containing 50 μg/ml kanamycin sulfate (Amresco, Solon, OH). *S*. *pyogenes* strains were grown in Bacto Todd Hewitt Broth (Becton, Dickinson and Company, Sparks, MD) supplemented with 2% Bacto yeast extract (THY medium) at 37°C or Brain Heart Infusion media (BHI) (Himedia Laboratories, India) at 37°C with the addition of the appropriate antibiotics (Sigma); growth was determined by monitoring the absorbance at 600 nm. Strain SF370SmR is a streptomycin resistant derivative of strain SF370, containing a spontaneous mutation in the *rpsL* gene [19]. No differences between SF370 and the streptomycin resistant derivative were observed with respect to growth rate or in the various biological assays employed [19]. Chemically defined media (CDM) was prepared as described in the literature [20].

**Table 1:** Strains, plasmid, and oligonucleotide primers used in this work.

**Strains**	**Genotype/Relevant characteristics**	**Reference**
SF370	*emm1* (contains prophages ϕSF370.1, ϕSF370.2, ϕSF370.3, and SpyCIM1)	[1, 14, 15]
SF370SmR	Spontaneous streptomycin resistant derivative of SF370	[19]
CEM1KRΔ *Spy2136*	SF370SmR Δ*Spy2136*::(*aacA-aphD) / (rpsL* ^*WT*^ *))*	[19]
CEM1Δ4	SF370SmRΔSpyCIM1; cured of SpyCIM1	[19]
MGAS5005	*emm1* (SpyCI-free M1 strain)	[21]
K56	*emm12* (SpyCI-free M12 strain)	[22]
**Plasmids**	**Description**	
pFWKR	Counter-selection vector with janus cassette, (*aacA-aphD) / (rpsL* ^*WT*^ *))* between MCS for phage elimination	[19]
pFWKR- *Spy2136*	pFWKR with flanking regions of *Spy2136* for inactivation of SpyCIM1 primase gene	[19]
**PCR Primers**	**Sequence**	**Product size**
mutSL-L	5' AATCGCCAGT TCCTGATGTC	1361 bp
mutSL-R	5' GGGCTGCTGA TGATTTGATT	
int4-L	5’ GTCGCTGTCT CATTTGATAG AGCTT	401 bp
int4-R	5’ CCAACAAGGA GTATTGCTAG GGC	
**qRT-PCR Primers**	**Sequence**	**Product size**
nga_RT-L	5' ACGTTAGCCG CAAATACCAC	89 bp
nga_RT-R	5' GCTTGTAACG TGGGAAGCTC	
slo-RT-F	5' AAGCTCCGCC ACTCTTTGTGA	103 bp
slo-RT-R	5' GCACTAAAGG CCGCTTCAAC 3'	
norA_RT-L	5' AAACGACGAC CAAACACCTC	102 bp
norA_RT-R	5' TCAGACTAGC CAGGCAGGAT	
emm RT-L	5' GCAAAACTAA GAGCTGGAAA	139 bp
emm RT-R	5' TAGTTTCCTT CATTGGTGCT	
speB RT-L	5' AGCAGTTGCA GTAGCAACACAT	141 bp
speB-RT-R	5' CTCCTTGATT CAAAAGGCATTC	
hasB_RT-L	5' TCCTCAAACG CTAATTGAAGC	92 bp
hasB_RT-R	5' CCCGCTCTTC TAAGACGTTG	
16S_rRNA-L	5’ AGCGTTGTCC GGATTTATTG	126 bp
16S_rRNA-R	5’ CACTCTCCCC TTCTGCACTC	

### Elimination of SpyCIM1 from *S*. *pyogenes* SF370SmR

A two-step phage counter-selection method using a Janus cassette was used to derive isolates that lost SpyCIM1 from the genome of SF370SmR; the complete details are described in Euler *et al*. [19]. In this strategy, the initial introduction of the Janus cassette results in the loss of streptomycin resistance and the acquisition of kanamycin resistance through replacement of the SpyCIM1 primase gene. Loss of the primase leads to instability of SpyCIM1 following excision, which promotes loss of this element. Curing of SpyCIM1 is then detected by a loss of kanamycin resistance with a concomitant restoration of streptomycin resistance. The Sm^R^ / Kan^S^ mutants identified following this process were then verified for the complete loss of the SpyCIM1 by PCR (using primers mutSL-L and mutSL-R to amplify the SpyCIM1 attachment site), Southern blot hybridization analysis (with SpyCIM1 specific gene probes), DNA sequence analysis, and PFGE analysis [19]. The resulting mutant strain, CEM1Δ4, no longer contained the integrated SpyCIM1 DNA in the streptococcal chromosome; further, the loss of SpyCIM1 restored the precise *mutS-mutL* junction seen in SpyCI-free strains of *S*. *pyogenes*.

### Chromosomal DNA Isolation, Sanger sequencing, and analysis

Isolation of streptococcal DNA was performed as previously reported [15, 23]. For isolation of stationary phase genomic DNA, a single colony of each *S*. *pyogenes* strain was used to inoculate 5 ml of fresh THY or BHI broth, which were allowed to grow >16 hours at 37°C in 5% CO_2_ before DNA isolation unless stated otherwise. Automated DNA sequencing was performed at the University of Oklahoma Health Sciences Center Laboratory for Genomics and Bioinformatics. Prior to sequencing, PCR products were treated with shrimp alkaline phosphatase and exonuclease I by incubation at 37°C for 60 min, followed by inactivation of the enzymes by heating at 85°C for 15 min or cleaned with the Wizard SV Gel and PCR Clean-Up System (Promega, Madison, WI). Sequencing was performed using the same primers that were used for PCR. PCR and Quantitative real-time PCR (qRT-PCR) were done as previously described [15, 16], using the primers listed in Table 1.

### Southern blot analysis

DNA was isolated from strains SF370SmR, CEM1Δ4, MGAS5005, and K56. Following digestion with HindIII, the generated fragments were separated on a 0.8% agarose gel with 0.5X tris-borate-EDTA (TBE) buffer and transferred to nylon membrane using standard methods [24]. A probe covering the SpyCIM1-free junction between mutS and mutL in strain MGAS5005 was prepared by PCR amplification of this region using primers MutSL-L and MutSL-R and the DIG-[11]-dUTP PCR labeling kit from Roche Life Science (Indianapolis, IN), following the manufacturer’s recommended protocol. Hybridization, washing, and detection of the probe bound to the blot was done also following the recommended protocol.

### Determination of the spontaneous mutation rate

A fluctuation test based on the Luria and Delbrück assay [25, 26] was used to determine the spontaneous mutation rates of *S*. *pyogenes* strains as previously described [15]. Briefly, THY broth was inoculated with an isolated colony of the strain to be tested and incubated overnight at 37°C. The overnight culture was diluted 1:10^4^ into 41 ml fresh THY broth such that the final cell density is <1000 cells/ml. The culture was dispensed as 1 ml aliquots into 31 sterile culture tubes. The cultures were grown for 24 hours at 37°C to achieve maximum cell density. One culture was used to determine total CFU by serial dilutions plated on THY agar, while the remainder were each mixed with 3.0 ml melted 0.6% agar in water (45°C) and overlaid onto THY agar plates supplemented with 2 μg/ml ciprofloxacin. After the agar overlay hardened, the plates were incubated at 37°C for >48 hours to allow appearance of antibiotic resistant mutants. The mutation rate with confidence limits was calculated for each strain using the software package *ft* (P.D. Sniegowski, Univ. Pennsylvania [27]). The data collected was averaged to determine mutation rate in mutations/generation (μ). Each determination was done at least three times.

### Sensitivity of strains to UV irradiation

The sensitivity of *S*. *pyogenes* strains to killing by UV irradiation from a calibrated 254 nm germicidal lamp (120 μW/cm^2^) was done as previously described [15]. Serial dilutions (ten-fold) of cells were exposed to UV light for up to two minutes in 30 second increments, in a darkened room to prevent photoreactivation, and were subsequently incubated overnight at 37°C to allow appearance of survivors. UV irradiation assays were conducted a minimum of three times per strain to confirm results.

### Growth inhibition by ethidium bromide

The resistance of strain SF370SmR or CEM1Δ4 was done using a modification of a previously reported method [28]. An overnight culture of *S*. *pyogenes* grown in THY broth at 37°C was diluted in fresh media to an absorbance at 600 nm of ~0.05. Incubation was continued at 37°C until A_600 nm_ = 0.3, and then the culture was diluted as before into fresh media containing 0, 1, 1.5, 2.5, or 5 μM ethidium bromide. The concentration of the ethidium bromide stock was determined using the extinction coefficient (ϵ) at 480 nm = 5680 M^-1^ cm^-1^ [29]. Each culture was grown as ten replicates (200 μl each) in a Bioscreen-C Automated Growth Curve Analysis System (Growth Curves US, Piscataway, NJ) at 37°C without shaking. The absorbance at 600 nm for each culture was measured every 15 minutes for a total of 24 hours. The 3 replicates of each condition where averaged and normalized to the cultures not treated with EtBr using the software package Graphpad Prism 4 (GraphPad Prism Software, La Jolla, CA).

### Ethyl methanesulfonate (EMS) mutagenesis

EMS mutagenesis was done using a modification of a previously described method [30]. THY broth (5 ml) was inoculated with a single colony of SF370SmR or CEM1Δ4 and incubated overnight at 37°C. The overnight culture was diluted into fresh THY (1:20 dilution) and grown at 37°C for two hours. A portion (0.5 ml) was spread on a THY agar plate containing 2 μg/ml ciprofloxacin and allowed to dry for 30 minutes. The EMS stock solution (1.145 g/ml; Sigma-Aldrich, St. Louis, MO) was diluted into sterile water to give a final working solution of 1 μg/ml; 15 μl of this solution was spotted on a sterile paper disk and placed in the middle of the agar plate that had been spread with the *S*. *pyogenes* strain. For a control, a paper disk that been spotted with distilled water was placed on a parallel agar plate for each strain. After overnight incubation at 37°C, the appearance of ciprofloxacin resistant mutants were tabulated.

### Global transcriptional analysis

Overnight cultures of SF370SmR and CEM1Δ4 were diluted 1:20 in filter sterilized (0.22μ) THY broth. The cultures were grown at 37°C or at 39°C to early log phase (A_600_ ~0.25) or to late log phase (A_600_ ~0.55). The cells were harvested by centrifugation, resuspended in 1 ml of RNA*Later* (Ambion #AM7021, Life Technologies, Grand Island, NY, U.S.A.) and stored at 4°C. To process the cells for RNA extraction, the cells stored in RNA*Later* were collected by centrifugation (8000xg for 5 min. at 4°C), and the pellets were resuspended in 200μL of RNA Storage Solution (1 mM Sodium Citrate, pH 6.4). The streptococci were lysed by incubation for 5 min at room temperature with PlyC Lysin (provided by V.A. Fischetti). All subsequent steps were performed at 4°C. RNA was extracted using Trizol Reagent (Life Technologies) and chloroform, using the manufacturer’s recommended protocol. The RNA pellets were dissolved in water (Molecular Grade Water, G-Biosciences #786–293, St. Louis, MO, U.S.A.) and quantified using a Nanodrop 1000 Spectrophotometer (Thermo Fisher Scientific Inc, Wilmington, DE, U.S.A.). The RNA samples were stored at -75°C until needed for further processing. Before RNA sequencing analysis, 16S and 23S ribosomal RNA was removed the samples by using a Microb*Express* kit (Ambion #AM1905) following the manufacturer’s protocol. The quality of the enriched mRNA samples was confirmed using an Agilent 2100 BioAnalyzer (Agilent Technologies, Inc., Santa Clara, CA, U.S.A.). The samples were converted to cDNA using Superscript II (Invitrogen, Carlsbad, CA) and random hexamer priming by following the manufacturer’s protocol. Library construction and whole transcriptome analysis was done at the Laboratory for Molecular Biology and Cytometry Research core facility at the University of Oklahoma Health Sciences Center using an Illumina MiSeq Next-Generation Sequencer and following the manufacturer’s recommended protocols (Illumina, Inc., San Diego, CA). Sequence reads were referenced to the *S*. *pyogenes* SF370 genome, and analysis was done using the software package GeneSifter (Geospiza, Inc. Seattle, WA). A likelihood ratio test was used to calculate the ratio of SF370SmR RNA to CEM1Δ4 RNA for each gene in the SF370 Genbank annotation. A Benjamini and Hochberg correction was applied to the data. The GEO accession for the datasets is record GSE75633. For mapping of mRNA and determination of polycistronic operons, the software package Rockhopper (http://cs.wellesley.edu/~btjaden/Rockhopper/) was used [31].

## Results

### Elimination of SpyCIM1 from the genome of strain SF370

**Fig 1:**
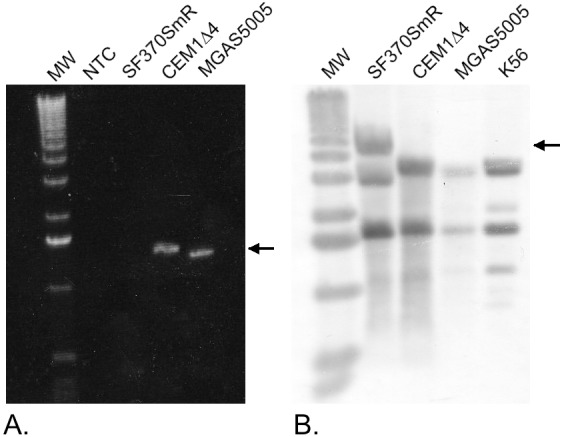
Elimination of SpyCIM1 (ϕ370.4) from its host strain. **A.** The prophage-free *attB* region can be amplified from a SpyCI-free M1 strain MGAS5005 and from SpyCIM1 cured strain CEM1Δ4, but not from the parental SF370SmR strain, where this region is interrupted by the presence of the 13.5 kb chromosomal island in the stationary phase cells. The arrow indicates the size of the expected 1361 bp PCR product following the loss of SpyCIM1. Lane 1 is a no template PCR control. **B.** Probing of a Southern blot of *Hin*dIII digested chromosomal DNA with a *mutS-mutL* probe showed SF370SmR has an altered pattern due to the presence of the integrated SpyCIM1 chromosome (indicated by the arrow), compared to CEM1Δ4, which has an identical hybridization pattern with SpyCIM1-free strains MGAS5005 and K56. MW: Kilobase molecular weight marker (Life Technologies, Grand Island, NY).

Our previous studies showed that SpyCIM1 controls a growth-dependent mutator phenotype in the *S*. *pyogenes* M1 serotype SF370 through a dynamic process of excision and re-integration into a unique site in DNA MMR gene *mutL* (S1 Fig). These studies suggested that having SpyCIM1 was associated with an increased mutation rate as well as sensitivity to lipophilic antimicrobials, UV irradiation, and DNA methylating agents [15, 16]. To confirm these results we chose to eliminate this chromosomal island and create an isogenic strain that would be wild type for the MMR operon. A two-step phage counter selection method was developed to derive isolates that had lost the 13.5 Kbp SpyCIM1 from the genome of SF370; this process has been described in detail elsewhere [19]. The initial step in the elimination process was the selection for a spontaneous streptomycin resistant derivative of SF370. This strain, SF370SmR, was used for all subsequent studies that compared it to the isogenic mutant, CEM1Δ4, cured of SpyCIM1. As shown in Fig 1, the loss of SpyCIM1 from the MMR operon could be confirmed by PCR and Southern blot. DNA was isolated from cells after overnight incubation at 37°C, a condition where we previously showed that SpyCIM1 would be integrated into the bacterial chromosome at *attB* [15]. The SpyCIM1 bacterial attachment site (*attB*) is the first 16 bases of the *mutL* ORF and begins 131 bases downstream of the *mutS* ORF. Under typical PCR conditions, this region can only be amplified if the chromosomal island is absent from the genome during logarithmic growth, when SpyCIM1 excises and replicates as an episome [15]. However, during stationary phase of growth the presence of the 13.5 Kbp of SpyCIM1 in the SF370SmR genome prevents normal amplification of this product by PCR. The removal of SpyCIM1 in the CEM1Δ4 mutant allowed for amplification of the *mutS*-*mutL* junction during both logarithmic and stationary phases of growth as occurs in SpyCIM1-free strains like MGAS5005 (Fig 1A). The loss of the chromosomal island also caused a change in the DNA hybridization pattern to this same region, with CEM1Δ4 having the same pattern as SpyCIM1-free strains (Fig 1B). The elimination of SpyCIM1 from the chromosome and the restoration of the normal DNA sequence observed in CEM1Δ4 was confirmed by DNA sequencing (not shown). Also, no SpyCIM1 genes could be amplified by PCR in strain CEM1Δ4, confirming that the element is lost and not translocated to a new site on the SF370SmR chromosome (not shown). Further, PFGE analysis of CEM1Δ4 digested genomic DNA showed no aberrant genomic DNA rearrangements were caused by the loss of SpyCIM1 [19].

### Elimination of SpyCIM1 reverses the mutator phenotype

**Fig 2:**
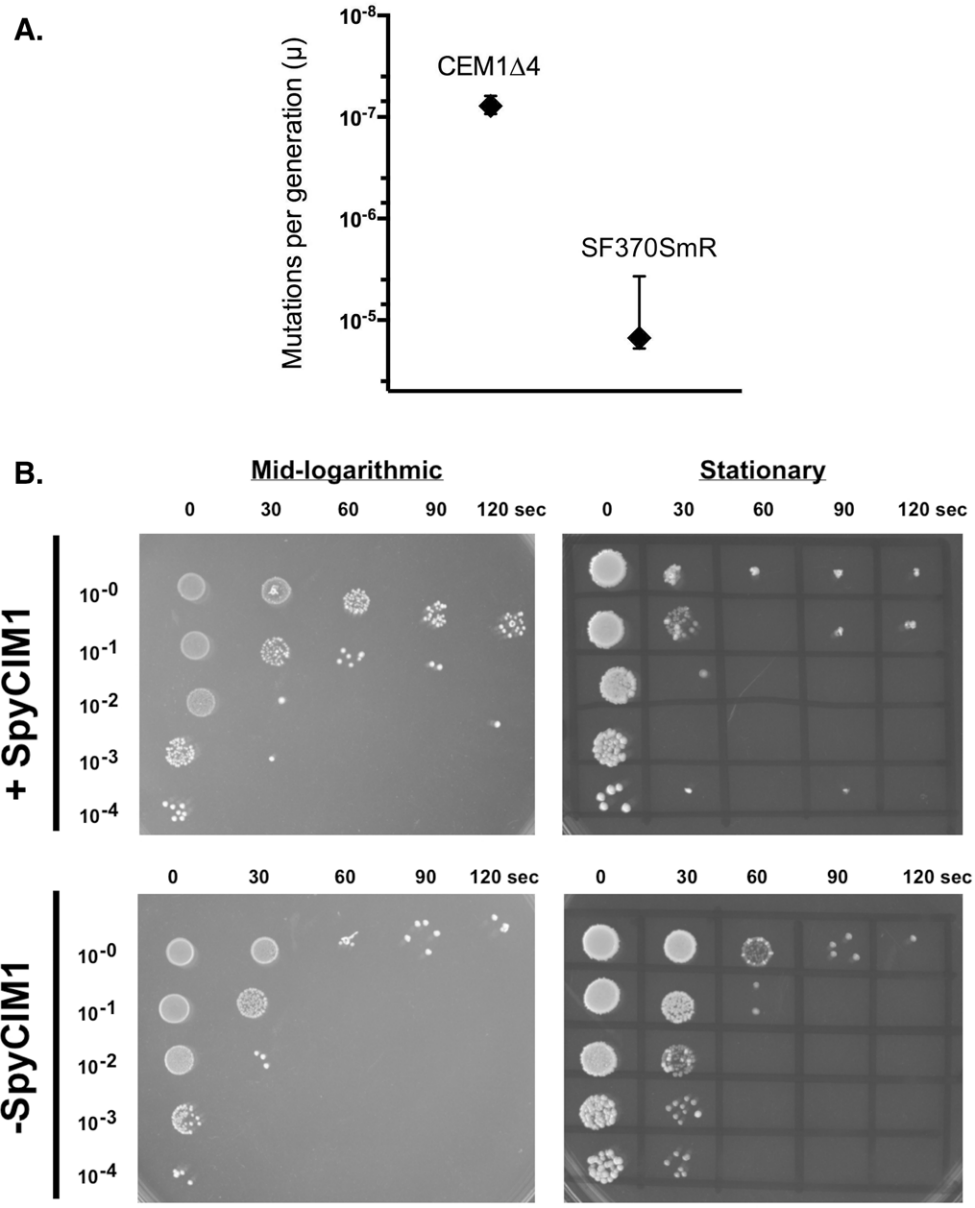
A. The loss of SPyCIM1 reverses of the mutator phenotype in SF370SmR. The rate of spontaneous mutation to ciprofloxacin resistance for isogenic strains CEM1Δ4 (SpyCIM1^-^) and SF370SmR (SpyCIM1^+^) was determined by fluctuation test, and the rate of mutations per generation (μ) was calculated. For each strain, 30 parallel cultures were established with ~1,000 CFU/culture, grown for 24 h at 37°C, and plated individually on selective media. After 48 to 96 h of incubation, colonies were enumerated, and mutation rates with 95% confidence limits were calculated using the maximum likelihood estimation technique as implemented by the program *ft* [27, 34, 35]. SpyCIM1-free strain CEM1Δ4 showed a 200-fold reduction in the rate of spontaneous mutation as compared to SF370SmR. B. Enhanced resistance to UV irradiation following loss of SpyCIM1. Ten-fold dilutions of strains SF370SmR and CEM1Δ4, which were growing in either logarithmic or stationary phase, were spotted onto an agar plate and exposed to 254 nm UV light (120 μW/cm^2^) for 0 to 120 sec. At logarithmic phase, when SpyCIM1 is excised from the chromosome [15], there was little difference in UV sensitivity between the strains. However, at stationary phase when SpyCIM1 is integrated into *mutL*, SpyCIM1 containing strain SF370SmR showed >100-fold more killing than isogenic strain CEM1Δ4, which lacks this element and is consistent with the restoration of *ruvA* expression. The protocol was performed in a darkened room to prevent photoreactivation. In the figure, the time in seconds (0–120) and the dilution factor of the cells are shown on the x-axis and y-axis, respectively.

We previously demonstrated that the presence of SpyCIM1 correlated with a mutator phenotype that was evidenced by an increase in the spontaneous mutation rate and an increase in sensitivity to UV irradiation, due to the inhibition of *mutL* and *ruvA* expression, respectively [15]. For these studies, the appearance of spontaneous resistance to ciprofloxacin was used to determine these rates since resistance results from a limited number of specific single nucleotide changes in *gyrA* or *parC* [32, 33] and thus provides a good readout for observing spontaneous point mutations. As shown in Fig 2, the elimination of SpyCIM1 from SF370SmR caused a reversal of both mutator phenotypes. Using a modified Luria and Delbrück fluctuation test, the spontaneous mutation rate (mutations per generation) for parental strain SF370SmR was determined to be 1.6 X10^-5^, a value similar to our previous estimates [15, 16]. Elimination of SpyCIM1 resulted in a 200-fold decrease in the mutation rate (Fig 2A), similar to *S*. *pyogenes* strains lacking SpyCIM1, which ranged from 10^−9^ to 10^−10^ mutations per generation in our previous studies [15, 16].

In similar fashion, the loss of SpyCIM1 resulted in an increased resistance to killing by UV irradiation, reflecting the restoration of *ruvA* expression (Fig 2B). Comparing the survival of CEM1Δ4 to SF370SmR shows that SF370SmR has between 10^2^ and 10^3^ fewer survivors in the stationary phase for a given irradiation time. Interestingly, sporadic survivors were seen in SF370SmR at higher cell dilutions but not in CEM1Δ4 following longer irradiation times (>1 min), perhaps resulting from the mutator phenotype of strain SF370SmR (Fig 2A). The appearance of these isolated survivors in only the SpyCIM1^+^ strain, SF370SmR, was observed in every replicate of this experiment (not shown). By contrast, no clear difference is seen between the two strains at mid-logarithmic growth when SpyCIM1 is extrachromosomal. Interestingly, mid-logarithmic SF370SmR cells appear more resistant to UV irradiation at the longer exposures, a phenomenon that may be related to the global transcriptional changes described below. However, at stationary phase the loss of *ruvA* function appears dominant and clearly differentiates the phenotype of the two strains.

**Fig 3:**
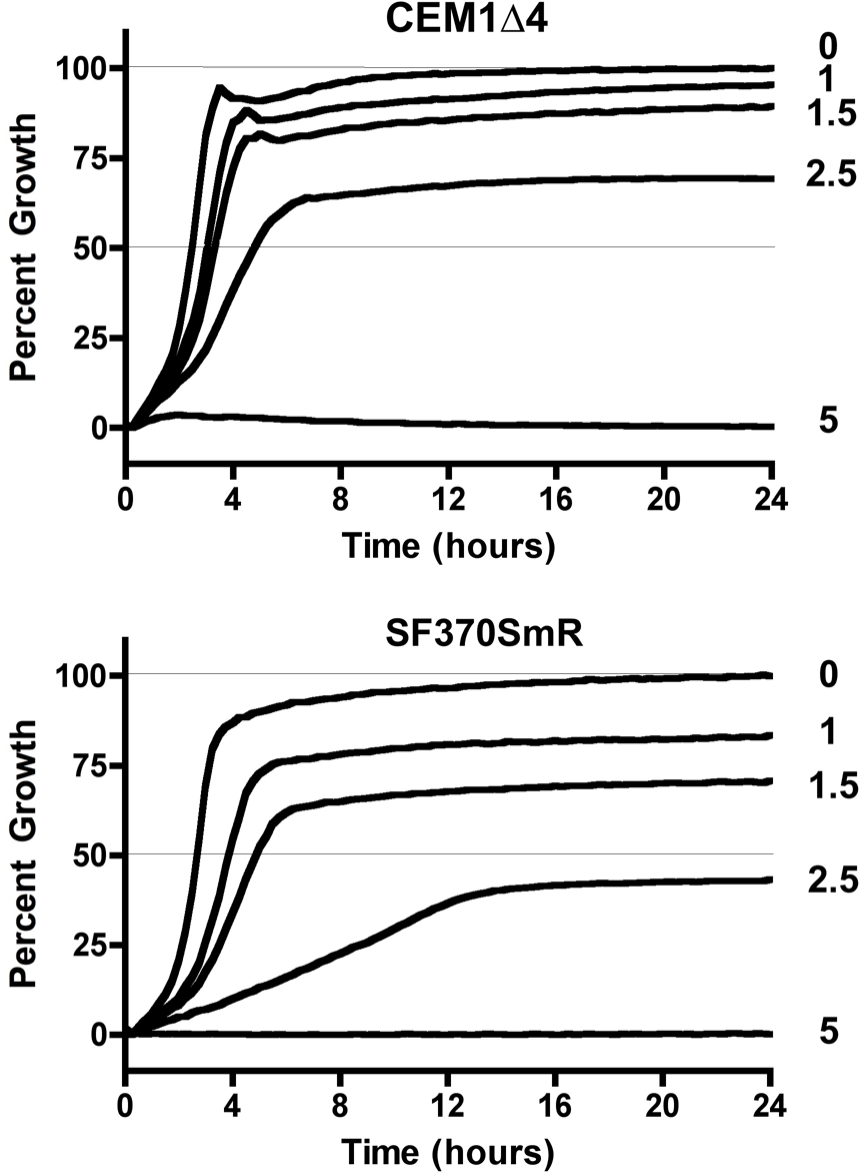
Resistance to ethidium bromide in *S*. *pyogenes* with and without SpyCIM1. Strains SF370SmR (lower panel) and its SpyCIM1 cured derivative CEM1Δ4 (upper panel) were grown at 37°C in THY broth with increasing concentrations of ethidium bromide (0, 1, 1.5, 2.5, and 5 μM). Each culture was grown in ten replicates, and growth was monitored every 15 minutes for 24 hours by the absorbance of the cultures at 600 nm. The data is presented as percentage of maximum growth observed for each strain without EtBr. Error bars, which were uniformly very small, are not shown for clarity of presentation. The numbers by each line indicate the concentration (μM) of EtBr in each culture.

The *lmrP* gene is a member of the operon interrupted by the presence of SpyCIM1. The encoded protein is a member of the major facilitator superfamily (MFS) of drug efflux proteins and has a hydropathy profile that predicts LmrP to be an integral membrane protein with 12 transmembrane regions [36]. A homolog of this gene is found in *Lactococcus lactis*, providing resistance to ethidium bromide and other antimicrobial compounds [37, 38]. Strains SF370SmR and CEM1Δ4 were grown in the presence of increasing concentrations of EtBr (0 to 5 μM) over a 24-hour period, and cell growth was monitored by the absorbance of the culture at 600 nm. In the absence of EtBr, both strains achieved >85% of the culture’s maximum density after 4 hours (Fig 3) and similar absolute culture densities (not shown). Similarly, neither strain grew appreciably at concentrations of 5 μM EtBr. In the SpyCIM1-free strain CEM1Δ4, the addition of EtBr up to 1.5 μM reduced the final density of the culture only by 10%, and while 2.5 μM EtBr resulted in a substantial reduction in growth, the culture still achieved a density of 70% of the untreated cells. By contrast, when compared to CEM1Δ4, strain SF370SmR showed a marked increase in sensitivity to EtBr. Treatment with 1.0 μM resulted in nearly 20% growth reduction, and cultures treated with 2.5 μM achieved a 60% growth reduction compared to untreated cells. Furthermore, the rate of growth of SF370SmR was markedly slowed under these conditions, requiring over 12 hours to achieve maximum density. Thus, the removal of SpyCIM1 from SF370SmR to allow constitutive expression of *lmr*P resulted in a marked increase in resistance to EtBr.

**Fig 4:**
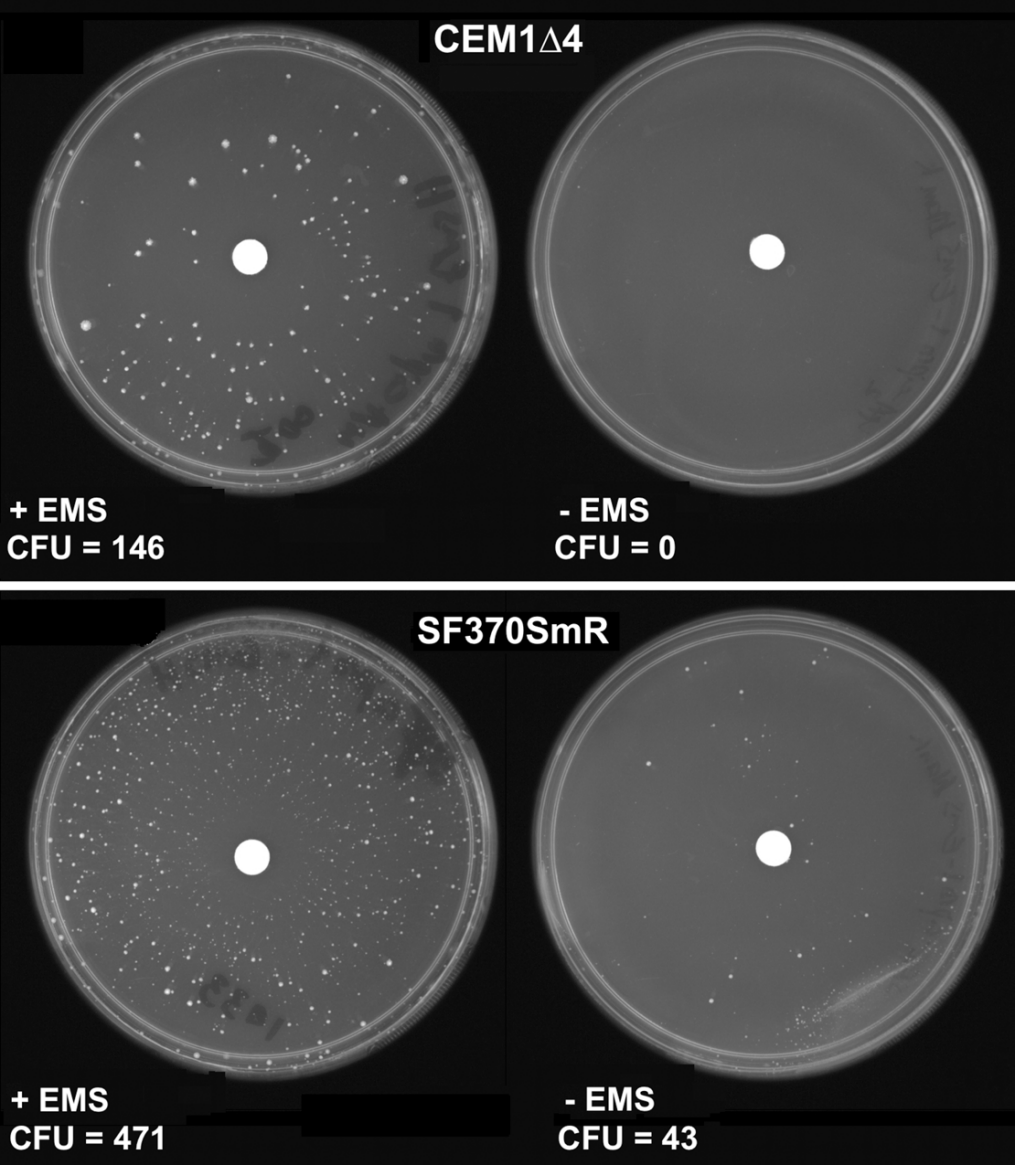
SpyCIM1 enhances the appearance of mutants following EMS treatment. Strains SF370SmR and CEM1Δ4 were spread on media plates containing ciprofloxacin (3 μg/ml). A paper disk containing either 15 ng EMS or sterile water was placed in the center of the plate, which was then incubated overnight at 37°C. The appearance of ciprofloxacin resistant mutants was observed in strain SF370SmR, even in the absence of EMS treatment (bottom right); under the same conditions strain CEM1Δ4 produced no resistant mutants (top right). Both strains produced ciprofloxacin resistant mutants following EMS treatment, but SF370SmR (bottom left) produced >3X as many resistant colonies as did SpyCIM1-free CEM1Δ4 (top left).

In *E*. *coli*, two 3-methyladenine-DNA glycosylases are present in the genome, encoded by *alkA* and *tag* [39]. These glycosylases are key components of base excision repair (BER), correcting a variety of alkylated bases that might lead to mutations; as seen with the mutagen EMS, which alkylates the O^6^ of guanine, leading to the formation O^6^-methylguanine causing G∙C → A∙T transitions [40, 41]. While no homolog has been identified in *S*. *pyogenes* to a known Gram-negative or -positive 3-meA DNA glycosylase II (*alkA*), a homolog of *tag* is located in the *S*. *pyogenes* MMR operon that would be controlled by SpyCIM1. Thus, the *S*. *pyogenes tag* gene product may play an essential role in the repair of alkylated bases, and its inhibition by SpyCIM1 could lead to an increased sensitivity to EMS or other DNA alkylating mutagens, resulting in an increased appearance of spontaneous mutations. Strains SF370SmR and CEM1Δ4 were treated with EMS to observe the appearance of chemically induced mutations leading to ciprofloxacin resistance. As shown in Fig 4, elimination of SpyCIM1 results in ~3-fold decrease in the appearance of spontaneous ciprofloxacin resistance in strain CEM1Δ4 after EMS treatment. Remarkably, even in the absence of EMS, SF370SmR still produced a few dozen spontaneous resistant mutants while CEM1Δ4 produced none. These studies show the increased sensitivity to induced mutagenesis by alkylating agents as well as the general mutator phenotype conferred by the presence of SpyCIM1.

### SpyCIM1 alters global transcription patterns in *S*. *pyogenes*

**Fig 5:**
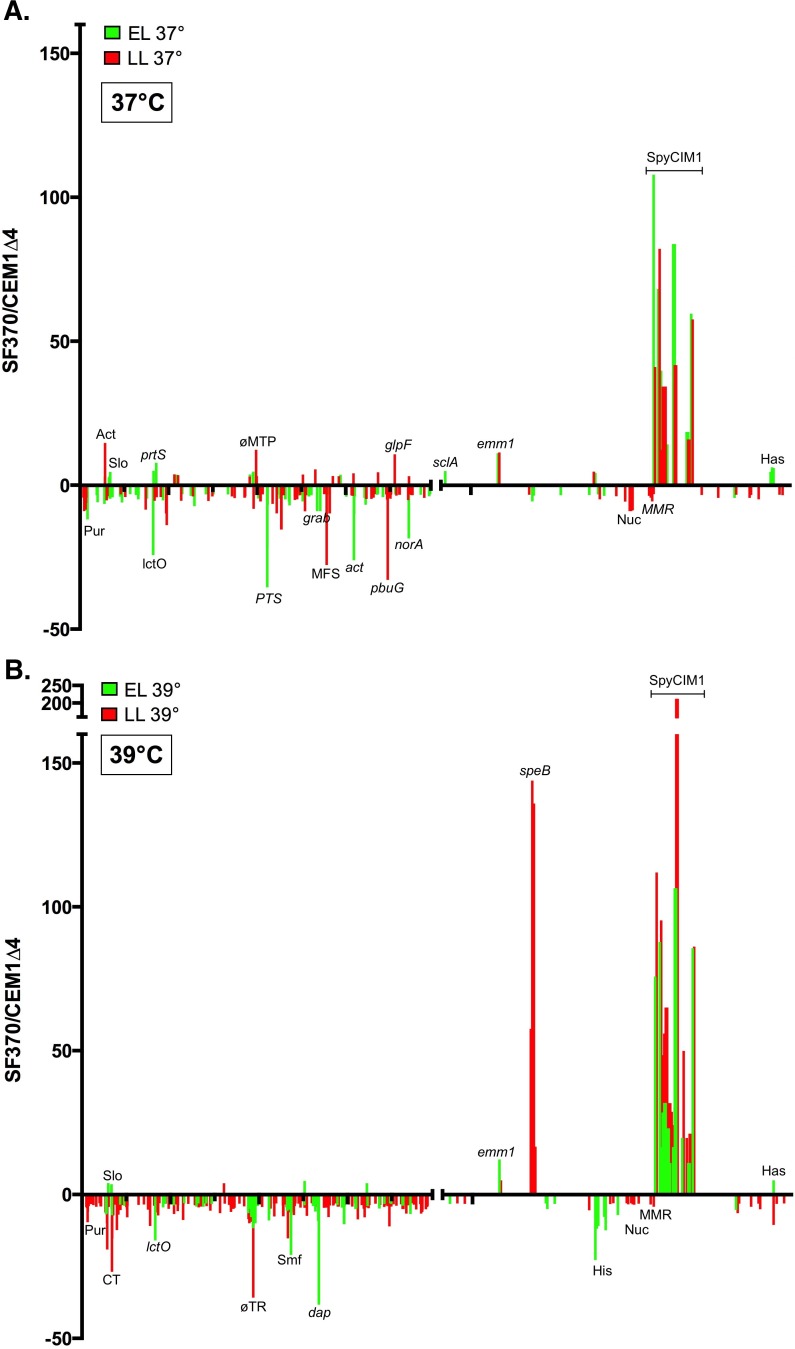
Elimination of SpyCIM1 alters global transcription patterns. RNA was isolated from SF370SmR or CEM1Δ4 at the onset of logarithmic growth (Early Log) or immediately before the cells entered stationary phase (Late Log); the cells were either grown at 37°C (**panel A**) or 39°C (**panel B**). Samples were then analyzed by RNA sequencing (RNA-Seq). In the ratio of gene expression from SF370SmR compared to CEM1Δ4 for values greater than 3 or less than -3 are plotted against the corresponding gene identification number from the SF370SmR annotation [1]. The MMR operon showed that no difference in expression was observed between the strains during early log phase but that the transcription of these genes was inhibited in SF370SmR at late log phase, in agreement with previous studies [15]; an expanded view of this region is shown in S3 Fig. Notable genes whose expression was altered in SF370SmR or CEM1Δ4 are identified. The ticks on the X-axis mark every 500 genes. Legend: Act–acetate CoA-transferase operon; *act*–acetyltransferase Spy154; *adhA*–alcohol dehydrogenase; *citM*—Mg2+/citrate complex transporter; *cspR* - 23S rRNA methyltransferase; CT- conserved transmembrane protein Spy0169; *dap*—diaminopimelate epimerase; *emm1* –M protein; *glfP*—glycerol uptake facilitator; *grab*—protein G-like alpha2-macroglobulin-binding protein; *has*–hyaluronic acid capsule operon; His–histidine catabolism operon; Hyp–gene encoding a protein of unknown function; *lctO*–lactate oxidase; MFS–uncharacterized major facilitator family protein (Spy1392); MMR–MMR operon; ϕMTP–phage major tail protein; *norA*—antibiotic resistance protein NorA; Nuc–nucleotide interconversions operon; *pbuG*—guanine-hypoxanthine permease; *pncA*—pyrazinamidase/nicotinamidase; *prtS*—cell envelope proteinase PrtS; PTS—mannose/fructose PTS system operon; *pur*–purine biosynthesis operon; sclA—collagen-like surface protein SclA; *slo*–streptolysin O operon; SpyCIM1 –phage-like CI. The identification of the SpyCIM1 genes is described in S1 Fig and S1 Table.

The studies presented here as well as our previous work argue that the expression of the MMR operon is controlled by the integrative state of SpyCIM1. Therefore, the global transcription patterns of SF370SmR and CEM1Δ4 were determined to observe whether the SpyCIM1 molecular switch might have an impact upon gene expression elsewhere in the *S*. *pyogenes* genome. Overnight growth of the two strains in CDM at 37°C suggested that their cell surface properties differed since SF370SmR had a “clumpy” phenotype (typical of high M protein surface expression) [42] while CEM1Δ4 did not (S2 Fig). RNA was isolated from SF370SmR and CEM1Δ4 at the initiation of logarithmic growth (early log phase; EL) and again late in logarithmic growth just before the cells entered stationary phase (late log phase; LL). High-throughput next generation RNA sequencing (RNA-Seq) was used to map and quantify the transcriptomes of these strains at those growth stages (Fig 5, Table 2, and S2–S6 Tables). The expression of the MMR operon was found to be the same in both strains at EL, in agreement with previous studies that demonstrated that SpyCIM1 excises from the chromosome and replicates as an episome at this time [15]. By LL the expression of the downstream MMR operon genes were depressed in SF370SmR by the re-integration of SpyCIM1, again in agreement with previous results. Analysis of the transcripts identified by RNA-seq allowed construction of a transcriptional map of SpyCIM1 (S4 Fig).

**Table 2:** Notable genes or operons whose transcription are altered by the presence of SpyCIM1. The numbers in parenthesis are the fold induction or repression in transcription of strain SF370SmR as compared to CEM1Δ4. For operons, the induction or repression is the average fold change for the affected genes. A likelihood ratio test was used to calculate the ratio of SF370SmR RNA to CEM1Δ4 RNA for each gene in the SF370 Genbank annotation. A Benjamini and Hochberg correction was applied to the data using the software package GeneSifter. Ratios greater than 3 or less than -3 are reported. Data are taken from S2 Table.

	**Early Logarithmic**		**Late Logarithmic**	
	**37°C**	**39°C**	**37°C**	**39°C**
**Induced** ^1^	M-protein (+11.1)	M-protein (+11.4)	M-protein (+12.2)	M-protein (+5.0)
	Capsule operon HasABC (+6)	Capsule operon HasABC (+3)	Major facilitator family protein NorA (+3.1)	Protease SpeB (+143.9)
	Collagen-like surface protein SclA (+4.9)		Transcriptional regulator Rgg3 (+3.8)	
	Streptolysin O operon (+3.0)			
	Chemokine protease ScpC (C5a peptidase family) (+5.0)			
	**Early Logarithmic**		**Late Logarithmic**	
	**37°C**	**39°C**	**37°C**	**39°C**
**Repressed**	Arginine deiminase operon (-10.1)	Arginine deiminase operon (-4.9)	Purine biosynthesis operon (-7.0)	Purine biosynthesis operon (-4.7)
	67 kDa Myosin-crossreactive streptococcal antigen (-5.3)	67 kDa Myosin-crossreactive streptococcal antigen (-3.2)	Guanine-hypoxanthine permease (-33)	Guanine-hypoxanthine permease (-11)
	L-lactate oxidase LctO (-24.3)	L-lactate oxidase LctO (-15.9)	Acetate CoA-transferase operon (-14.5)	Capsule operon HasABC (-6)
	Multidrug resistance protein NorA homolog (-18.5)	Multidrug resistance protein NorA homolog (-6.8)	Malic enzyme (MaeE) (-9.8)	Smf family DNA processing protein (-15)
	Thioesterase family protein Spy1339 (-9)	Thioesterase family protein Spy1339 (-38)		Nicotinate-nucleotide pyrophosphorylase (-12)
	Transcriptional regulator RopB/Rgg1 (-3.6)	D-lactate dehydrogenase (-5.2)		Transcriptional regulator ComR (Rgg4) (-4.1)
	Alcohol dehydrogenase (-11.9)	Histidine catabolism operon (-13)		Ferrichrome ABC transporter permease operon (-7.4)
	ComYA (-5.9)			
	Protein G-like alpha 2M-binding protein (GRAB) (-9)			
	PTS system mannose/fructose family transporter operon (-18)			
	Acetyltransferase Spy1546 (-26)			

Surprisingly, the presence of SpyCIM1 correlates with global transcriptional changes in *S*. *pyogenes* with genes showing differential basal expression in EL, LL, or both (Fig 5A, Table 2, and S2 Table). While some of these global changes may be related to the differential expression of the MMR operon, other changes must map directly back to SpyCIM1 since in EL strains SF370SmR and CEM1Δ4 differ only by the presence or absence of the extrachromosomal form of this chromosomal island. Several key virulence genes have altered expression in SF370SmR; perhaps the most striking is the >11-fold higher expression of the major antiphagocytic M1 protein gene (*emm1*) in SF370SmR as compared to CEM1Δ4 in both EL and LL. The hyaluronic capsule operon (*hasABC*) is also more highly expressed in SF370SmR during EL as well as the virulence operon encoding NADase (*nga*) and streptolysin O (*slo*). Other SF370SmR genes were strongly depressed in EL as compared to CEM1Δ4, particularly the genes for the homolog of the MFS antibiotic resistance pump *norA* [43] and for lactate oxidase (*lctO*). The down-regulation of *lctO* may promote survival in SF370SmR by preventing lethal self-intoxication by H_2_O_2_ production [44]. Interestingly, *norA*, although depressed in EL, is more highly expressed in SF370SmR in LL. Inhibition of NAD-dependent malic enzyme MaeE has recently been linked to both *S*. *pyogenes* survival in low pH and to increased virulence [45], and this gene has nearly a 10-fold LL reduction in SF370SmR. Many changes related to metabolic pathways are also observed in the transcriptome of SF370SmR. The mannose/fructose phosphotransferase (PTS) operon (Spy1057-Spy1060) is down regulated in SF370SmR in EL, while an oxalate:formate antiporter (Spy1392), which is another MFS efflux protein, is strongly down regulated in LL (by ~27-fold). Many other metabolic changes are seen in SF370SmR, including depression of purine metabolism; the details are presented in S3 and S4 Tables.

Transcriptional patterns of cells grown at 39°C were examined also (Fig 5B, Table 2 and S2 Table). This temperature was chosen to mimic alterations in transcription that might result from growth in a febrile human. The M-protein (*emm1*) remained more highly expressed in SF370SmR regardless of growth phase or culture temperature. Similarly, the capsule operon (*hasABC*) remained more highly expressed in EL while L-lactate oxidase (*lctO*), the arginine deiminase operon, the multidrug resistance protein *norA* homolog, and the 67 kDa myosin-crossreactive streptococcal antigen were all decreased in expression in SF370SmR during EL at either 37° or 39°C. Other genes were altered in expression in a temperature dependent fashion, including several global transcriptional regulators. Transcriptional regulator Rgg3 has increased expression at 37°C during late log growth but not at 39°C while RopB (Rgg1) and ComR (Rgg4) are repressed by the presence SpyCIM1 in early log growth at 37°C and late log growth at 39°C, respectively. The most dramatic shift in expression, however, occurs in the gene for SpeB, which is increased over 140-fold in SF370SmR over CEM1Δ4 at 39°C in late log cells; qRT-PCR from the same cDNA preparations confirmed this difference in expression between the strains (S7 Table). To determine if this induction in *speB* expression was reproducible, three independent cultures of both strains were grown at 39°C, and samples were removed for RNA isolation at EL and LL as before. An additional sample was taken one hour after LL. The cDNA from these RNA samples were used as template for qRT-PCR to determine the expression of *speB* and other genes showing differential expression (S8 Table). In agreement with the RNA-seq data, this analysis confirmed the enhanced expression of *speB* as well as the differential expression of *nga*, *slo*, *norA*, *emm*, and *hasB* (S8 Table). Taken together, these results argue that SpyCIM1 encodes some mechanism that alters the transcription program of SF370SmR in a way that extends beyond only regulation of the MMR operon.

## Discussion

In these studies, we demonstrate how the elimination of SpyCIM1 from the chromosome of *S*. *pyogenes* strain SF370SmR resulted in a decreased spontaneous mutation rate and increased resistance to ethidium bromide, UV irradiation, and EMS mutagenesis, by allowing constitutive expression of the MMR operon. Our previous studies relied on inference to demonstrate the impact of SpyCIM1 on the host phenotype through the comparison of unrelated streptococcal isolates [15, 16]. The present studies provide direct evidence for the SpyCIM1 associated mutator phenotype using isogenic strains that differed only by the presence of this chromosomal island. Further, these studies show that elimination of SpyCIM1 alters global transcription in the host streptococcus cell, and that these changes could potentially reduce virulence. Thus, the data provide important new information on the emerging field of Gram-positive phage-like chromosomal islands that alter the host bacterium’s phenotype.

The chromosomal island SpyCIM1 is a dynamically active phage-like element that alternates between chromosomal and episomal states, which mediates phenotypic changes upon the host bacterium. The penetrance of these phenotypes reflects the relative proportion of the population that has SpyCIM1 in the excised or integrated form. Therefore, an observed phenotype might vary over time as environmental or physiological conditions could favor the integrated or episomal state of SpyCIM1. An example of such changes was illustrated in Fig 4, where the resistance to UV irradiation was essentially the same in cells having or lacking SpyCIM1 during logarithmic growth when SpyCIM1 was episomal. However, a distinct phenotype became apparent when the cells were in stationary phase and SpyCIM1 was integrated into the bacterial chromosome. It is probable that SpyCIM1 mobilization during normal cell division results from an event that is linked to the cell cycle, but the process also appears to be inducible by DNA damage since mitomycin C treatment promotes SpyCIM1 excision [15]. Little is known about the details of SOS repair and its induction in *S*. *pyogenes*; indeed, no homolog of LexA is readily identifiable. Therefore, the molecular control of SpyCIM1 mobilization either triggered by SOS repair or by normal growth remains to be discovered.

Global transcriptional analysis showed the basal expression of numerous genes is altered by the presence of SpyCIM1. While notable differences could be seen between SF370SmR and CEM1Δ4 in both EL and LL cultures, the role of SpyCIM1 is clearly evident in the EL comparisons when the two strains differ only by the presence of the extrachromosomal chromosomal island in SF370SmR (Fig 5 and S2 Table). Thus, the transcriptional differences in EL do not result from transcriptional inhibition of the MMR operon but from the presence of SpyCIM1 encoded gene products in the cell.

The global gene expression changes associated with SpyCIM1 appear to either occur independently of target subsets of known *S*. *pyogenes* regulatory networks. For example, the Mga regulon includes a number of virulence genes such as the M protein (*emm1*), C5a peptidase (*scpA*), secreted inhibitor of complement (*sic*) and streptococcal collagen-like protein (*scl1/sclA*) [46]. In EL, the expression of the *emm1* is 11-fold and *sclA* ~5-fold higher in SF370SmR, respectively, as compared to CEM1Δ4. By contrast, the C5a peptidase and Sic mRNAs are expressed equally in both strains, as is the expression of Mga itself. Therefore, the differences in gene expression seen between the two strains appears to step outside of the characterized regulatory networks in group A streptococci [46], suggesting that some SpyCIM1 encoded factor is responsible. SpyCIM1 contains a number of genes encoding proteins with a predicted helix-turn-helix (HTH) structure (S1 Table). The HTH products of genes Spy2125 and Spy2126 probably function as the repressor and antirepressor for SpyCIM1 integration and excision, given their position and orientation in the genome that is analogous to the lysogeny module of many prophages; however, there are other SpyCIM1 genes encoding HTH proteins (Spy2127, Spy2134, and Spy2145) whose biological role is currently unknown. Mga binding sites are reported to be diverse, having only 13.4% identity [47], and so some other DNA binding proteins such as the ones encoded by SpyCIM1 could potentially target only a sub-set of this regulon.

Many of these transcriptional changes may enhance virulence or survival. The increase in M protein and capsule expression would be antiphagocytic while the repression of arginine deiminase [48] and L-lactate oxidase [44, 49] at 37°C in EL may enhance virulence or prevent autointoxication by hydrogen peroxide production, respectively. The expression of the exotoxin SpeB is influenced by a number of *S*. *pyogenes* transcriptional regulators [50], and so, it is not surprising that it and its co-transcribed genes are stimulated in expression under some conditions (LL and 39°C), although the degree of induction seen was striking (>140-fold in SF370SmR as compared to CEM1Δ4; Table 2); qRT-PCR confirmed this difference in expression from identical samples between the strains (S7 Table). This induction is specific for 39°C since no differences in expression between the two strains was observed for SpeB at 37°C. Further, while the degree of induction was not as high as in the original experiment, growth of replicate cultures of both strains at 39°C confirmed the enhanced expression of *speB* (average fold induction in SF370SmR over CEM1Δ4 = 18.3 ± 11.9), and the differential expression of the other genes analyzed (S8 Table). Since it has been demonstrated previously that the induction of *speB* is sensitive to the growth state of the cell as well as environmental conditions [51–53], it is possible that SpyCIM1, either directly or indirectly, alters the timing of *speB* expression in LL at 39°C, leading to earlier expression of the gene in SF370SmR. Sampling of RNA from these cultures one hour later eliminates this difference, and indeed, CEM1Δ4 has about a ~2 fold greater expression of *speB* by this stage of growth (S8 Table). Future studies will be needed to clarify the differences in kinetics of *speB* expression between SF370SmR and CEM1Δ4 in fine detail.

In *E*. *coli*, it has been reported that select RNA chaperones and a cold shock protein are overexpressed to compensate for a mutator phenotype [54]. None of the potential DEAD box helicase RNA chaperones (Spy0288, Spy1369, Spy1659, Spy1837, and Spy1415) were altered in expression by the loss of SpyCIM1; however, cold-inducible RNA chaperone and antiterminator protein Csp (Spy2077) was inhibited 5.5-fold in SF370SmR at 39°C in LL. Thus, the inhibition of Csp may further enhance the SpyCIM1-associated mutator under these conditions. These shifts in gene expression, which appear to cut across a number of known regulatory networks, may improve fitness of the SpyCIM1 host cell. The potential benefits to a host bacterium following phage toxigenic conversion have been proposed many times [55–58], but the lack of such an identifiable virulence factor in SpyCIM1 led to the suggestion that it was a prophage remnant [1, 14]. While homologs of SpyCIM1 have been frequently observed in subsequent *S*. *pyogenes* genomes, their descriptions as a nonfunctional cryptic element remained until their role in regulating the MMR operon was discovered [15]. The results presented here argue that the impact of SpyCI on streptococcal survival and virulence extends well beyond controlling this operon.

Several qualifications must be considered in interpreting the transcriptional analysis data. The populations of cells are not synchronous so many differences seen may be the average expression of genes from cells that may be in different stages of growth; this situation is particularly important in SF370SmR where the population is undoubtedly a mixture of cells having the integrated or episomal form of SpyCIM1 [15]. Further, the altered expression of some genes may not be a direct result of some SpyCIM1-encoded gene product but result from the altered expression of some other host gene or regulatory network by the chromosomal island. Other differences in expression between cells grown at 37°C or 39°C may be due to enhanced or accelerated mRNA turnover at the higher temperature. Some changes, however, must map to altered promoter function caused either by a SpyCIM1 encoded product (protein or RNA) or perturbation of some *S*. *pyogenes* regulatory network.

Altered expression was observed in some genes encoding known regulatory proteins, which could contribute to the differing phenotypes. Comparing the expression of SF370SmR to CEM1Δ4, *ropB* (*rgg1*; Spy2042) was inhibited >3X at 37°C and EL, *rgg3* (Spy0533 [20]) was increased >4X at 37°C and LL, and *comR* (*rgg4*; Spy0037) was inhibited >4X at 39°C and LL. Interestingly, the RNA-seq data showed that RofA-like gene *ralp3* [59], which has a frameshift in SF370SmR, was expressed as a truncated transcript encoding amino acids 411 through 461 of the complete protein; the significance of this expression is unknown. Some of these shifts in regulation may influence horizontal transfer in group A streptococci. Although our understanding of group A streptococcal transformation is at an early stage [60], the inhibition of ComR (Rgg4) expression in SF370SmR at 39°C and LL might inhibit both competence and biofilm formation as recently demonstrated [60]. Perhaps related to controlling horizontal transfer, the Smf family DNA processing protein/DNA polymerase sliding clamp subunit (Spy1163) is also inhibited under the same conditions, and this protein functions as a transformation-dedicated DNA loader for RecA in *S*. *pneumoniae* [61]. Genes ComEC and ComEA, encoding homologs of the DNA translocation machinery channel protein in *S*. *pneumoniae* [62] are similarly inhibited by these conditions. Since Marks and coworkers found that group A streptococcal competence was highest at 34°C and decreased when the temperature was raised to 37°C [60], it is perhaps not surprising that a further increase to 39°C would down-regulate any associated genes. However, our transcriptional studies show that the presence of SpyCIM1 appears to enhance this limitation to horizontal transfer at elevated temperatures.

The regulation of the gene expression in Gram-positive bacteria by small, phage-like chromosomal islands is an emerging theme in prokaryotic biology [18, 63], and the examples that have been identified so far modify host cell function in a variety of ways from toxigenic conversion to gene regulation. We have shown here the direct impact SpyCIM1 has upon the expression of the MMR operon in *S*. *pyogenes*, but many questions concerning this system remain to be answered. For example, the activation of SpyCIM1 excision appears to be linked to the onset of cell division [15], but the cellular signals that trigger this event as well as the subsequent molecular decision to re-integrate into *mutL* remain unidentified. However, the most interesting field for exploration is how SpyCI promotes host fitness or survival, particularly in natural human infections. The acquisition of a mutator phenotype appears to increase bacterial virulence under some circumstances as a recent study of base excision repair mutants in *Streptococcus mutans* demonstrated [64]. The dynamic nature of this chromosomal island may prove to be an essential component of its relationship with *S*. *pyogenes*. Since MMR deficient strains are frequently found in many bacterial species, it might be inferred that inactivation of this repair system alone should not destabilize a cell. However, the one example we have of a defective and permanently integrated SpyCI (in the M5 genome strain Manfredo) did not result in silencing the MMR operon but rather demonstrated rescue of the operon through the evolution of a cryptic promoter within the chromosomal island remnant [16]. This result suggests that rounds of MMR operon expression and silencing may prove to have strong selective value, allowing a phenotypic flexibility that is unobtainable with simple gene inactivation through mutation. Thus, the dynamic control of this repair system by SpyCIM1 is an ideal method for achieving the mutator phenotype while minimizing the risks associated with long-term hypermutability.

In spite of decades of antibiotic therapy, *S*. *pyogenes* has shown a remarkable ability to maintain its niche as a major human pathogen; it has the ability to infect, colonize, and rapidly adapt to multiple environments in the human body with different tissue tropisms, while periodically producing novel strains associated with severe disease outbreaks. The novel, switchable mutator phenotype coupled with global transcriptional changes conferred by the SpyCI element may prove to be an important factor contributing to the adaptability and the evolutionary robustness of this pathogen.

## Supporting Information

S1 Fig Model of SpyCIM1 regulation of the MMR operon in strain SF370. Phage-like chromosomal island SpyCIM1 regulates the expression of an operon containing genes *mutL*, *lmrP*, *ruvA*, and *tag* in response to cell growth [15, 16]. The presence of an integrated SpyCIM1 results in the cells adopting a mutator phenotype with regard to DNA mismatch repair (MMR), multiple drug efflux, Holliday junction resolution, and base excision repair. In early logarithmic phase, SpyCIM1 excises from the SF370 chromosome, restoring expression of *mutL* and the downstream genes (insert). As the cells approach stationary phase, SpyCIM1 re-integrates into *mutL*, silencing gene expression. This process of SpyCIM1 excision and integration causes the cells to alternate between a wild type and mutator phenotype. In the figure, transcriptionally active MMR operon genes are black while inactive ones are gray. The identification and potential function of the SpyCIM1 genes are detailed in S1 Table). Adapted from Nguyen and Scott [63], and used by permission.
(TIF)

S2 Fig Clumping phenotype of SF370SmR following growth in CDM. Strains SF370SmR and CEM1Δ4 were grown overnight at 37°C in CDM. Gentle shaking was used to simultaneously disperse each cell pellet, showing that SF370SmR had a clumping phenotype that CEM1Δ4 lacked. The cultures were streaked on blood agar to confirm that both were pure cultures of each starting strain.
(TIF)

S3 Fig Expanded view of SpyCIM1 and MMR operon RNA-seq transcription patterns at 37° and 39°C. The data are taken from Fig 5. The first gene in the MMR operon, *mutS*, is not shown since no differences in transcription were ever observed between SF370SmR and CEM1Δ4. Only differences in transcription ≥ ±3-fold are shown.
(TIF)

S4 Fig Transcriptional map of SpyCIM1. Transcriptome analysis of mRNA from SF370SmR allowed prediction of the messages encoded by SpyCIM1, including probable polycistronic ones. The genetic map of SpyCIM1 is shown flanked by the MMR operon genes. Row **A** below the genetic map shows the predicted mRNAs that match genes in the SF370SmR annotation. Row **B** shows small RNAs that were detected by RNA-Seq but which are not included in the Genbank annotation. The small RNA immediately upstream of the *int* mRNA encodes a small peptide with a transmembrane domain. Prediction was accomplished using the software package Rockhopper [31].
(TIF)

S1 Table The identification and probable function of SpyCIM1 ORFs. The protein IDs refer to the ORF numbers in the published SF370 genome [1]. Products are predicted by BLASTP homology of the encoded proteins to GenBank entries. Several additional ORFs have been identified since the original annotation was prepared.
(PDF)

S2 Table Transcriptome analysis of CEM1Δ4 compared to SF370SmR at early log (EL) and late log (LL) growth phases. A likelihood ratio test was used to calculate the ratio of SF370SmR RNA to CEM1Δ4 RNA for each gene in the SF370 Genbank annotation. A Benjamini and Hochberg correction was applied to the data using the software package GeneSifter. Ratios greater than 3 or less than -3 are reported and were used to create the plot in Fig 5.
(PDF)

S3 Table KEGGS analysis of the early log phase (EL) transcriptome comparison between CEM1Δ4 and SF370SmR grown at 37°C. The analysis was done using GeneSifter as above.
(PDF)

S4 Table KEGGS analysis of the late log phase (LL) transcriptome comparison between CEM1Δ4 and SF370SmR grown at 37°C. The analysis was done using GeneSifter as above.
(PDF)

S5 Table KEGGS analysis of the early log phase (EL) transcriptome comparison between CEM1Δ4 and SF370SmR grown at 39°C. The analysis was done using GeneSifter as above.
(PDF)

S6 Table KEGGS analysis of the late log phase (LL) transcriptome comparison between CEM1Δ4 and SF370SmR grown at 39°C. The analysis was done using GeneSifter as above.
(PDF)

S7 Table Quantitative real-time PCR (qRT-PCR) validation of RNA-seq transcriptional analysis. The cDNA preparations used for RNA-seq analysis were analyzed by qRT-PCR for comparing the expression of *nga*, *slo*, *norA*, *emm*1, *speB*, and *hasB* in SF370SmR to CEM1Δ4 as described in the Methods.
(PDF)

S8 Table Reproducibility of gene expression differences between SF370SmR and CEM1Δ4 at 39°C. Three independent cultures of SF370SmR and of CEM1Δ4 were grown at 39°C; samples were harvested for RNA isolation when the culture density (A_600 nm_) was 0.2 (**EL**) and again when the density was 0.5 (**LL**). An addition sample was harvested one-hour post LL (**Stationary**). After conversion of the RNA to cDNA, qRT-PCR was used to compare expression levels of the listed genes between SF370SmR and CEM1Δ4. Values are the average and standard deviation of the fold-difference between the two stains.
(PDF)
