# Supplementary figures and images for: Elimination of Chromosomal Island SpyCIM1 from Streptococcus pyogenes Strain SF370 Reverses the Mutator Phenotype and Alters Global Transcription
Source: PLoS One. 2015 Dec 23;10(12):e0145884. doi: 10.1371/journal.pone.0145884 (PMC4689407; doi:10.1371/journal.pone.0145884)

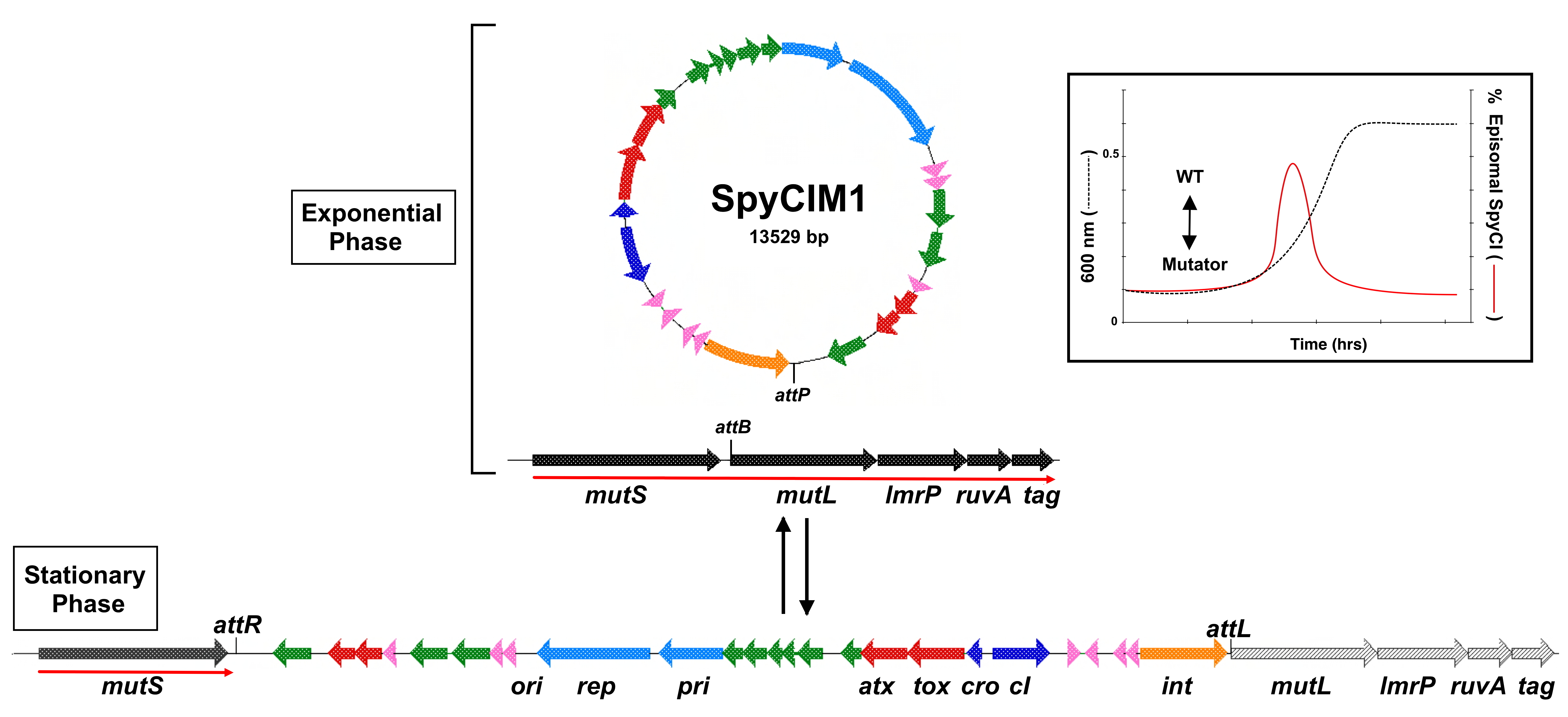

Supplement: S1 Fig — Phage-like chromosomal island SpyCIM1 regulates the expression of an operon containing genes mutL, lmrP, ruvA, and tag in response to cell growth [15, 16]. The presence of an integrated SpyCIM1 results in the cells adopting a mutator phenotype with regard to DNA mismatch repair (MMR), multiple drug efflux, Holliday junction resolution, and base excision repair. In early logarithmic phase, SpyCIM1 excises from the SF370 chromosome, restoring expression of mutL and the downstream genes (insert). As the cells approach stationary phase, SpyCIM1 re-integrates into mutL, silencing gene expression. This process of SpyCIM1 excision and integration causes the cells to alternate between a wild type and mutator phenotype. In the figure, transcriptionally active MMR operon genes are black while inactive ones are gray. The identification and potential function of the SpyCIM1 genes are detailed in S1 Table). Adapted from Nguyen and Scott [63], and used by permission. (TIF) [file pone.0145884.s001.tif]

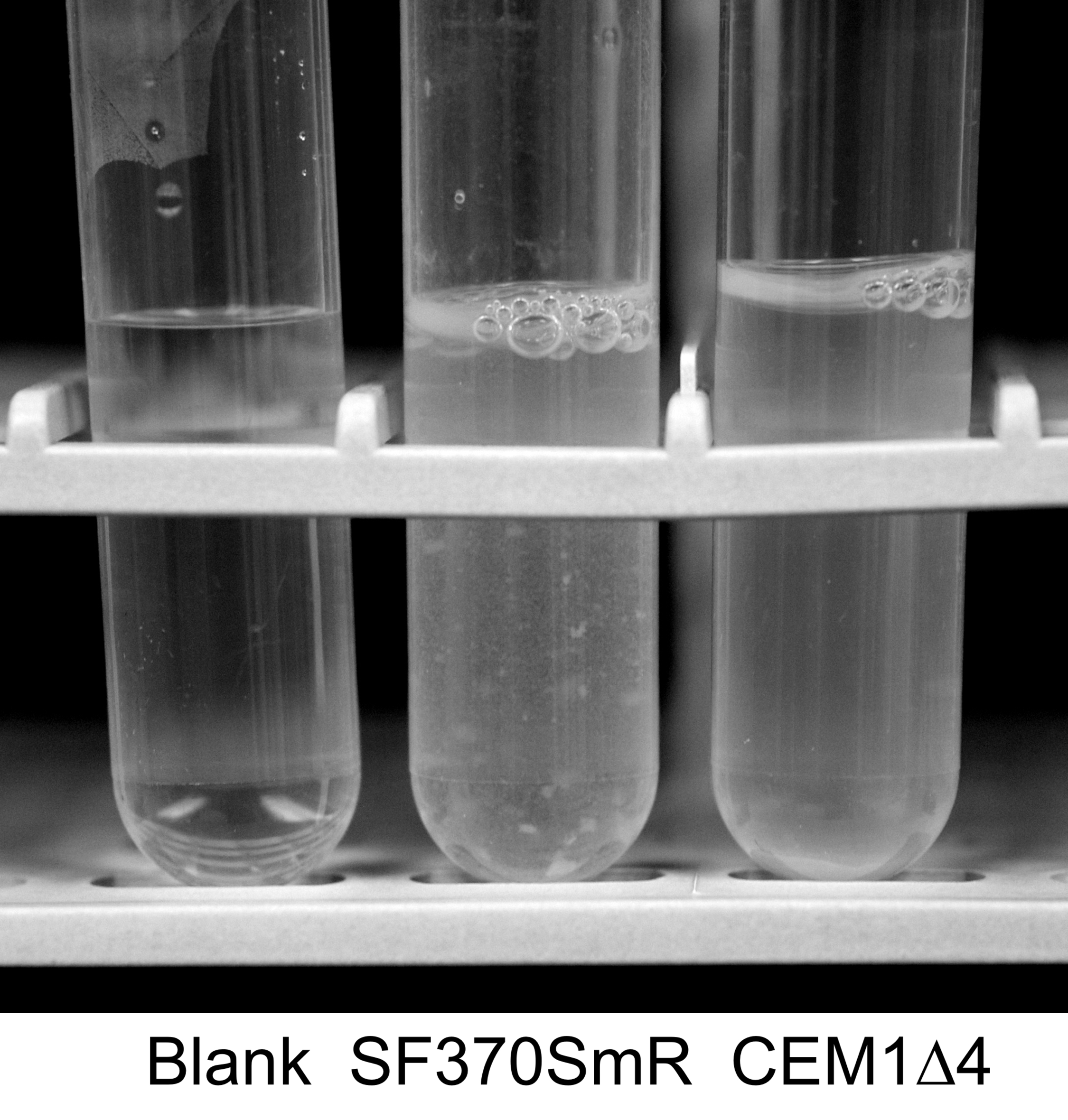

Supplement: S2 Fig — Strains SF370SmR and CEM1Δ4 were grown overnight at 37°C in CDM. Gentle shaking was used to simultaneously disperse each cell pellet, showing that SF370SmR had a clumping phenotype that CEM1Δ4 lacked. The cultures were streaked on blood agar to confirm that both were pure cultures of each starting strain. (TIF) [file pone.0145884.s002.tif]

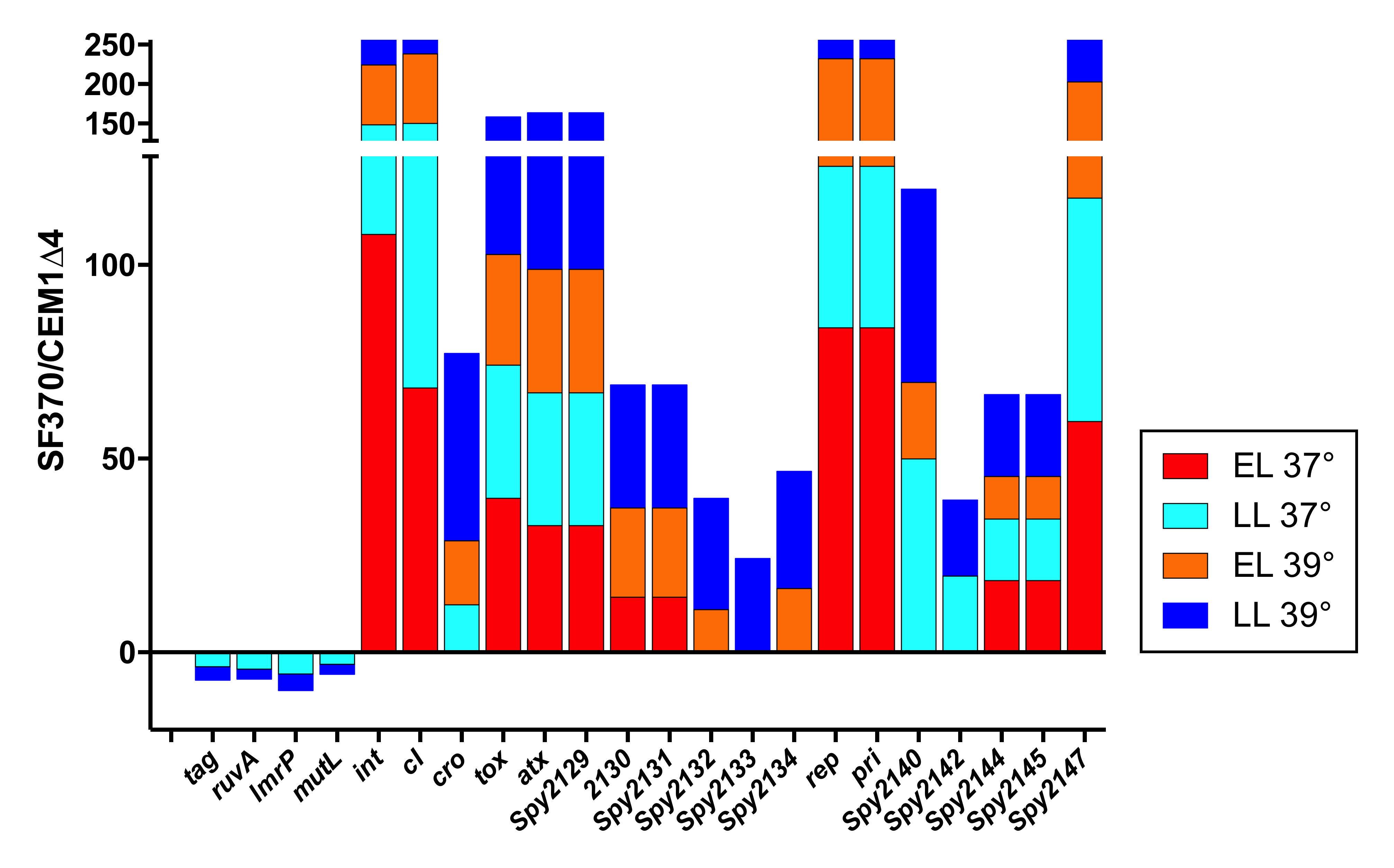

Supplement: S3 Fig — The data are taken from Fig 5. The first gene in the MMR operon, mutS, is not shown since no differences in transcription were ever observed between SF370SmR and CEM1Δ4. Only differences in transcription ≥ ±3-fold are shown. (TIF) [file pone.0145884.s003.tif]

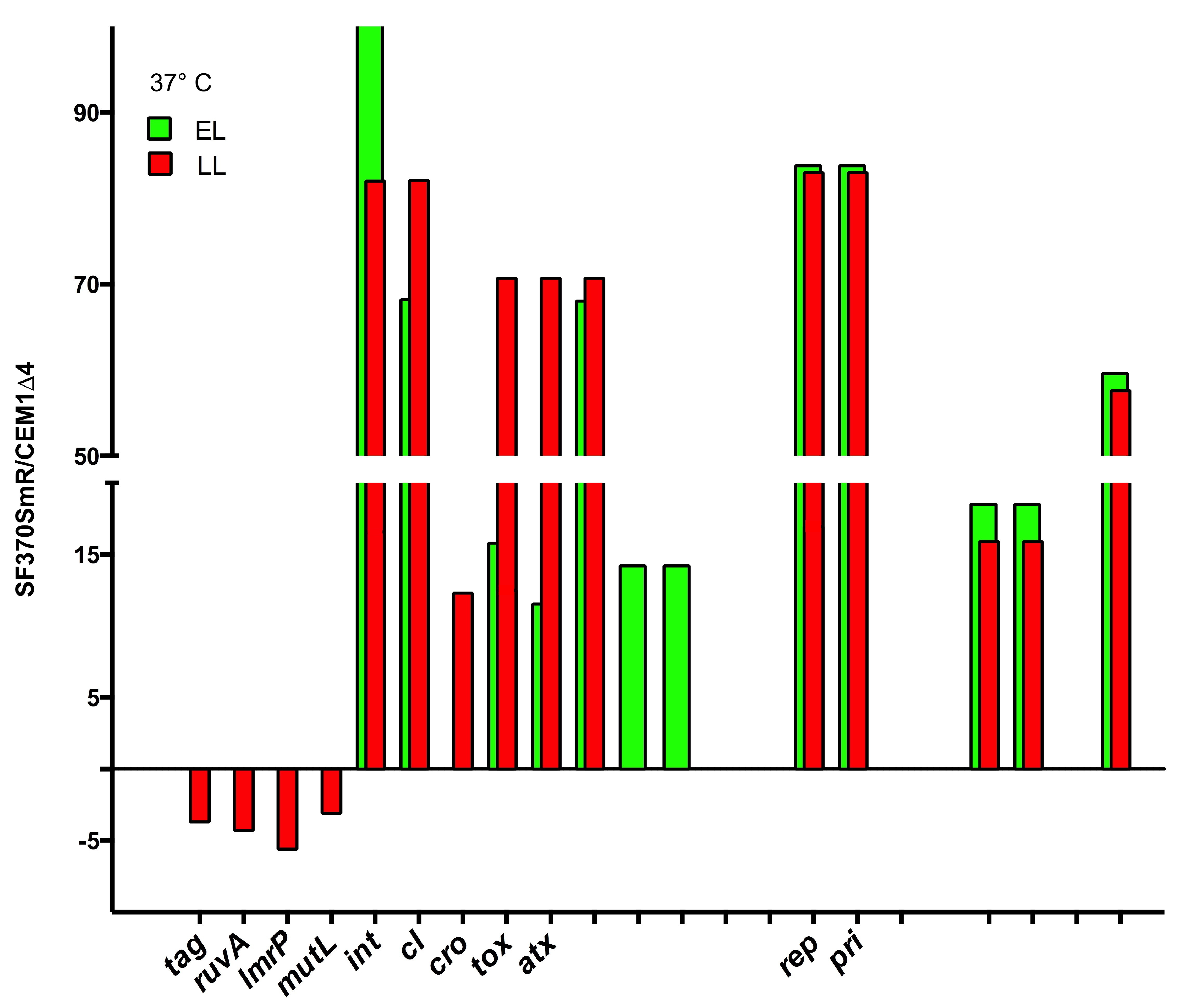

Supplement: S4 Fig — Transcriptome analysis of mRNA from SF370SmR allowed prediction of the messages encoded by SpyCIM1, including probable polycistronic ones. The genetic map of SpyCIM1 is shown flanked by the MMR operon genes. Row A below the genetic map shows the predicted mRNAs that match genes in the SF370SmR annotation. Row B shows small RNAs that were detected by RNA-Seq but which are not included in the Genbank annotation. The small RNA immediately upstream of the int mRNA encodes a small peptide with a transmembrane domain. Prediction was accomplished using the software package Rockhopper [31]. (TIF) [file pone.0145884.s004.tif]
