# Supplementary material for: Elimination of Chromosomal Island SpyCIM1 from Streptococcus pyogenes Strain SF370 Reverses the Mutator Phenotype and Alters Global Transcription
Source: PLoS One. 2015 Dec 23;10(12):e0145884. doi: 10.1371/journal.pone.0145884 (PMC4689407; doi:10.1371/journal.pone.0145884)
Supplement: S1 Table — The protein IDs refer to the ORF numbers in the published SF370 genome [1]. Products are predicted by BLASTP homology of the encoded proteins to GenBank entries. Several additional ORFs have been identified since the original annotation was prepared. (PDF) [file pone.0145884.s005.pdf]

S1 Table.

SpyCIM1 ORF	Protein ID	Predicted product	Gene	Coordinates	Comment
1	SPy2122	Integrase	*int*	1774028-1775170	
2				1775236-1775394	Not in Genbank annotation
3				1775425-1775565	Transmembrane domain peptide; not in Genbank annotation
4				1775783-1775941	Not in Genbank annotation
5	SPy2125	Repressor for immunity	*cI*	1776432-1777196	HTH motif
6	SPy2126	Anti-repressor	*cro*	1777350-1777553	HTH motif
7	SPy2127			1777590-1778354	HTH motif; BRO family, N-terminal domain protein
8	SPy2128			1778367-1778987	BRO family, N-terminal domain protein
9	SPy2129			1778990-1779256	
10	SPy2130			1779515-1779853	Homologous to *Lactobacillus plantarum* WCFS1 prophage Lp4 protein 11, DNA replication
11	Spy2131			1779843-1780061	
12	Spy2132			1780067-1780255	
13	Spy2133			1780270-1780596	
14	Spy2134			1780602-1780871	HTH motif
15	SPy2135	Replicase	*rep*	1780875-1781729	Contains homologous replication module to *Streptococcus thermophilus* plasmid pSt106 [53]
16	Spy2136	Primase	*pri*	1781701-1783386	
17				1783676-1783846	Not in Genbank annotation
18				1783855-1784025	Not in Genbank annotation
19	SPy2140			1784030-1784536	
20	SPy2142			1784613-1785098	
21				1785325-1785474	Not in Genbank annotation
22	Spy2144			1785505-1785864	
23	Spy2145			1785842-1786222	HTH motif
24	SPy2147			1786426-1786971	Contains a Gram-positive signal peptide
