# Supplementary material for: Elimination of Chromosomal Island SpyCIM1 from Streptococcus pyogenes Strain SF370 Reverses the Mutator Phenotype and Alters Global Transcription
Source: PLoS One. 2015 Dec 23;10(12):e0145884. doi: 10.1371/journal.pone.0145884 (PMC4689407; doi:10.1371/journal.pone.0145884)
Supplement: S2 Table — A likelihood ratio test was used to calculate the ratio of SF370SmR RNA to CEM1Δ4 RNA for each gene in the SF370 Genbank annotation. A Benjamini and Hochberg correction was applied to the data using the software package GeneSifter. Ratios greater than 3 or less than -3 are reported and were used to create the plot in Fig 5. (PDF) [file pone.0145884.s006.pdf]

S2 Table.

		37°C		39°C			
Gene Identifier[1]	Gene[2]	Ratio EL[3]	Ratio LL	Ratio EL	Ratio LL		Polycistronic mRNA
900379	Spy0019		-4.3			Putative secreted protein/amidase	
900384	Spy0024		-9.0		-4.3	Phosphoribosylaminoimidazole succinocarboxamide synthase PurC	
900385	Spy0025		-8.5		-3.1	Phosphoribosylformyl-glycinamidine synthase II	
900386	Spy0026		-8.0		-3.4	Amidophosphoribosyltransferase PurF	
900387	Spy0027		-7.4		-4.0	phosphoribosylformylglycinamide cyclo-ligase	
900388	Spy0028		-6.1		-4.7	Phosphoribosylglycinamide formyltransferase	
900389	Spy0031		-8.5		-9.6	Choline binding protein	
900390	Spy0032		-8.5		-6.0	Phosphoribosylamine-glycine ligase PurD	
900391	Spy0033		-6.2		-3.4	Phosphoribosylaminoimidazole carboxylase PurE	
900392	Spy0034		-6.2		-3.4	Phosphoribosylaminoimidazole carboxylase PurK	
900393	Spy0035		-4.4			Abortive infection protein AbiR	
900394	Spy0036		-4.1			Adenylosuccinate lyase PurB	
900395	Spy0037				-4.1	Transcription regulator ComR (Rgg4)	
900400	Spy0044	-11.9				Alcohol dehydrogenase AdhA	
900402	Spy0047				-3.3	30S ribosomal protein S10	
900412	Spy0060				-3.3	30S ribosomal protein S17	
900416	Spy0064				-3.7	30S ribosomal protein S14	
901998	Spy0080				-4.3	50S ribosomal protein L17	
900436	Spy0096	-3.5				Tyrosyl-tRNA synthetase TyrS	
900439	Spy0099				-3.0	DNA-directed RNA polymerase subunit beta	
900441	Spy0101	-5.9				Putative ABC transporter subunit ComYA	
900445	Spy0105				-4.1	Hypothetical protein	
900446	Spy0106				-4.1	Competence protein ComYD	
900447	Spy0107				-4.1	Competence protein ComG	
900464	Spy0133				-6.3	IS66 ORF2 like protein	
900465	Spy0135				-5.1	Sortase	
900467	Spy0137			-6.9	-6.6	Short-chain fatty acids transporter AtoE	
900468	Spy0139	-6.5		-6.6		LysR type transcriptional regulator	
900470	Spy0141	3.0	ND[4]	-10.0		Acetate CoA-transferase subunit subunit alpha	

900471	Spy0142	ND[4]	14.7	-19.1		Acetate CoA-transferase subunit beta
900473	SPy0145	-3.1				Hypothetical protein Yjgf
900474	Spy0145	-1.3*				PfoR-like transmembrane protein
900477	Spy0149			4.0		V-type ATP synthase subunit K NtpK
900478	Spy0150				-4.3	V-type ATP synthase subunit K NtpE
900479	Spy0151		-5.3			V-type Na+ ATP synthase subunit C
900481	Spy0155		-3.8			V-type ATP synthase subunit B
900484	Spy0159	-3.4				Hypothetical protein
900488	Spy0165	2.6*		2.3*		NAD glycohydrolase (NADase) Nga
900489	Spy0166	2.6*		3.1		NAD glycohydrolase inhibitor
900490	Spy0167	3.0		3.6		Streptolysin O (Slo)
900491	Spy0168	-4.6		-5.0	-5.0	Hypothetical protein
900492	Spy0169			-7.2	-26.9	Conserved transmembrane protein
900493	Spy0170			-2.9	-15.2	Conserved transmembrane protein
900494	Spy0171	4.6			-4.6	Hypothetical protein
900495	Spy0172	-4.7				Cystathione gamma synthase (CGS) MetB
900497	Spy0174	-3.2				PTS system ascorbate-specific transporter subunit IIC
900499	Spy0176				-7.3	PTS system 3-keto-L-gulonate specific transporter subunit IIA
900505	Spy0182	-4.2				L-ascorbate 6-phosphate lactonase
900507	Spy0184				-3.6	Glycine-betaine binding permease
900518	Spy0197				-12.3	Nicotinate-nucleotide pyrophosphorylase
900523	Spy0205				-7.0	Zinc finger protein
900529	Spy0216			-3.6	-5.4	Regulatory protein
900535	Spy0227	-4.0			-3.7	MarR family transcriptional regulator
900536	Spy0228	-2.8			-1.8	MarR family transcriptional regulator
900538	Spy0230		-3.2			ABC transporter ATP-binding protein
900543	Spy0238	-6.0		-3.9		Hypothetical protein
900551	Spy0250				-4.4	50S ribosomal protein L34
900553	Spy0252				-3.7	N-acetylneuraminate-binding protein
900554	Spy0254				-5.1	N-acetylneuraminate ABC transporter permease
900555	Spy0255		-4.1		-7.9	N-acetylneuraminate ABC transporter permease
900556	Spy0256				-4.0	Hypothetical protein
900580	Spy0285	-3.1				ABC transporter ATP-binding protein
900604	Spy0317	-3.4				ABC transporter substrate-binding protein
900605	Spy0319				-3.6	ABC transporter substrate-binding protein
900613	Spy0330	-4.9				Cytoplasmic membrane protein LemA

900614	Spy0331	-3.3				Heat shock protein HtpX
900626	Spy0349				-3.5	Transcription elongation factor GreA
900643	Spy0371		-8.5			23S rRNA methyltransferase
900644	Spy0373				-5.3	Riboflavin transporter
900645	Spy0374				-3.4	Phosphatidylglycerophosphatase B-like protein
900649	Spy0379	-4.7				Pyruvate formate-lyase activating protein
900652	Spy0383				-3.8	Ferrichrome ABC transporter permease
900653	Spy0384				-10.9	Ferrichrome ABC transporter permease
900667	Spy0407				-3.9	Hypothetical protein
900671	Spy0414	-24.3		-15.9		L-lactate oxidase LctO
900672	Spy0416	5.0			-3.9	C5a family peptidase; chemokine protease ScpC
900673	Spy0421		-5.4			Hypothetical membrane protein
900675	Spy0425		-3.9		-6.2	Ribonucleotide-diphosphate reductase subunit beta
900676	Spy0426		-3.7		-3.4	Ribonucleotide reductase stimulatory protein
900677	Spy0427		-3.7		-3.4	Ribonucleotide-diphosphate reductase subunit alpha
900679	Spy0430				-7.3	Hypothetical protein
900680	Spy0431	7.8				Hypothetical protein
900682	Spy0433		-4.2			Hypothetical protein
900685	Spy0437				-3.1	Hypothetical protein
900698	Spy0456		-4.1			ABC transporter permease
900701	Spy0459			-4.5		Hypothetical protein
900710	Spy0470	-5.3		-3.2		67 kDa Myosin-crossreactive streptococcal antigen
900711	Spy0471				-3.2	Phosphate starvation-induced protein
900722	Spy0486		-9.8			Hypothetical protein
900724	Spy0489		-13.9		-5.0	Hypothetical protein
900731	Spy0502				-3.1	Preprotein translocase subunit SecG
900736	Spy0507				-3.3	Permease
900737	Spy0508				-3.3	Permease
900747	Spy0519			-3.7	-6.8	ABC Transporter permease
900754	Spy0530				-3.5	Zn-dependent hydrolase VicX
900757	Spy0533		3.8			Positive transcriptional regulator Rgg3
900764	Spy0540				-5.8	Hypothetical protein
900765	Spy0542				-3.7	UDP-glucose 6-dehydrogenase
900769	Spy0546	3.6				Chromosome segregation ATPase
900770	Spy0547	3.6				Hypothetical protein

900771	Spy0549	3.6				Hypothetical protein
900774	Spy0553		3.5			Toxin-antitoxin system, antitoxin component, PHD family
900787	Spy0571		-5.4		-8.8	Transcription antiterminator LicT
900788	Spy0572				-3.4	PTS system beta-glucoside-specific transporter subunit IIABC
900792	Spy0578	-3.1		-3.0		Hypothetical membrane spanning protein
900809	Spy0600				-3.2	Thiamine transporter
900826	Spy0627	-3.7				LacI family transcriptional regulator
900834	Spy0637		-3.9		-3.5	Possible sugar-phosphate isomerase
900835	Spy0638				-3.7	2-dehydro-3-deoxygluconokinase
900842	Spy0646	-3.3				Possible sugar-phosphate isomerase
900843	Spy0647	-7.3		-4.2		Acetoin dehydrogenase
900853	Spy0658			-3.1	-4.5	Cro-like protein (Prophage 370.1)
900855	Spy0660				-3.9	Hypothetical protein (Prophage 370.1)
900856	Spy0661				-3.9	Hypothetical protein (Prophage 370.1)
900857	Spy0663				-3.9	Chromosome separation protein (Prophage 370.1)
900859	Spy0665				-3.5	Hypothetical protein (Prophage 370.1)
900860	Spy0666				-3.8	Hypothetical protein (Prophage 370.1)
900863	Spy0670				-4.2	Hypothetical protein (Prophage 370.1)
900864	Spy0671				-4.2	DNA replication protein (Protein 370.1)
900877	Spy0686			-4.3		Hypothetical protein (Prophage 370.1)
900878	Spy0688				-3.1	Major head protein (Prophage 370.1)
900890	Spy0702			-3.1		65 KD protein associated with hyaluronidase (Prophage 370.1)
900891	Spy0703				-4.0	Hypothetical protein (Prophage 370.1)
900897	Spy0712	-3.2				Phage mf2 DNase (Prophage 370.1)
900909	Spy0724				-3.8	50S ribosomal protein L19
900910	Spy0725		-5.5		-5.6	Transposase
900911	Spy0726				-3.1	DNA gyrase subunit B
900917	Spy0733			-3.3		Transposase
900929	Spy0747		-3.9			Predicted nuclease
900933	Spy0754	-3.2				ATP synthase F0F1 subunit C
900955	Spy0779				-6.5	30S ribosomal protein S21
900974	Spy0801				4.0	Ferredoxin
900979	Spy0806				-3.2	50S ribosomal protein L20
900997	Spy0830				-3.6	Bifunctional pyrimidine regulatory protein PyrR/uracil

						phosphoribosyltransferase
901002	Spy0836	-3.2				Transporter-like protein
901006	Spy0840				-4.1	30S ribosomal protein S16
901007	Spy0841				-6.1	RNA binding protein
901017	Spy0853				-5.0	Transcriptional repressor
901018	Spy0854				-5.0	Fructose-1-phosphate kinase
901019	Spy0855				-5.0	PTS system fructose-specific transporter subunit IIABC
901021	Spy0857		-3.9			Peptidoglycan hydrolase
901023	Spy0861				-4.6	IgG-degrading protease Mac-1
901030	Spy0872		-4.5			5'-nucleotidase
901047	Spy0889				-3.2	Ribose-5-phosphate isomerase A
901070	Spy0916				-4.1	Hypothetical protein
901081	Spy0928		-4.3			Similar to DNA replication protein
901082	Spy0929		-4.3			Endonuclease III
901085	Spy0932		-3.9			Oxidoreductase
901092	Spy0940			-6.5	-8.6	Hypothetical protein (prophage 370.2)
901095	Spy0944			-3.9	-5.3	Hypothetical protein (prophage 370.2)
901096	Spy0945			-5.0	-10.0	Cro/C1-type HTH DNA-binding domain protein repressor (prophage 370.2)
901097	Spy0946			-4.1	-7.2	P1-type antirepressor (prophage 370.2)
901098	Spy0947			-3.9		Hypothetical protein (prophage 370.2)
901099	Spy0948			-3.2	-5.1	Hypothetical protein (prophage 370.2)
901100	Spy0949				-5.6	Hypothetical protein (prophage 370.2)
901101	Spy0951			-3.5	-8.2	Excisionase (frameshift mutation) (prophage 370.2)
901102	Spy0952				-7.1	Hypothetical protein (prophage 370.2)
901103	Spy0953				-5.6	Hypothetical protein (prophage 370.2)
901104	Spy0954				-5.6	Hypothetical protein (prophage 370.2)
901105	Spy0956			-7.8	-7.4	Hypothetical protein (prophage 370.2)
901106	Spy0957				-6.2	Hypothetical protein (prophage 370.2)
901107	Spy0958		3.0	-3.7	-5.2	Phage recombination protein Bet (prophage 370.2)
901108	Spy0959			-3.9	-9.5	Hypothetical protein (prophage 370.2)
901109	Spy0960	3.7		-4.7	-5.7	Hypothetical protein (prophage 370.2)
901110	Spy0961				-7.7	Hypothetical protein (prophage

						370.2)
901111	Spy0962				-7.7	Hypothetical protein (prophage 370.2)
901112	Spy0963				-4.8	Hypothetical protein (prophage 370.2)
901113	Spy0965				-5.1	Hypothetical protein (prophage 370.2)
901114	Spy0967			-11.7	-35.9	ArpU family transcriptional regulator (prophage 370.2)
901115	Spy0968			-3.3	-3.1	Hypothetical protein (prophage 370.2)
901116	Spy0970			-7.8		Hypothetical protein (prophage 370.2)
901122	Spy0977	4.3				Hypothetical protein (prophage 370.2)
901124	Spy0979			-10.0		Hypothetical protein (prophage 370.2)
901125	Spy0980	4.7	-8.2	-7.3		Phage associated antirepressor (prophage 370.2)
901137	Spy0994		12.3	-4.0		Minor tail protein (prophage 370.2)
901138	Spy0995		3.1			Putative phage tail component, N-terminal domain protein (prophage 370.2)
901139	Spy0996		3.1			Phage endopeptidase (prophage 370.2)
901140	Spy0997		3.1			Phage associated hyaluronidase (prophage 370.2)
901146	Spy1007	-4.6				Exotoxin I (prophage 370.2)
901147	Spy1008	-3.1				Exotoxin H (prophage 370.2)
901152	Spy1016		-3.0			ABC transporter substrate-binding protein
901153	Spy1017				-4.3	Hypothetical protein
901154	Spy1018		-5.6			ABC transport protein permease
901155	Spy1019		-5.6			ABC transporter ATP-binding protein
901163	Spy1031		-3.2			Dihydrolipoamide dehydrogenase, component E3
901185	Spy1057	-35.5		-9.0		PTS system mannose/fructose family transporter subunit IIA
901186	Spy1058	-21.5		-6.9		PTS system mannose/fructose family transporter subunit IIB
901187	Spy1059	-6.8		-1.9*		PTS system mannose/fructose family transporter subunit IIC
901188	Spy1060	-6.8		-1.9*		PTS system mannose/fructose family transporter subunit IID
901193	Spy1065				-4.9	Acetyltransferase
901210	Spy1085				-3.1	Lantibiotic associated ABC transporter ATP-binding protein SrtF
901211	Spy1086				-7.6	Lantibiotic associated protein SrtE
901212	Spy1087		-6.5			Lantibiotic associated protein SrtG

901217	Spy1096				-3.1	Folyl-polyglutamate synthetase
901230	Spy1110		-9.8			NAD-dependent malic enzyme MaeE
901231	Spy1111			-5.2		Zinc-containing alcohol dehydrogenase
901232	Spy1113	-3.3				Acid phosphatase / phosphotransferase AphA
901246	Spy1128	-4.7				Phosphotransacetylase EutD
901248	Spy1131	-3.1				Na+ driven multidrug efflux pump
901251	Spy1135				-3.8	Guanosine 5'-monophosphate oxidoreductase
901252	Spy1136		-15.4		-7.5	Xanthine phosphoribosyltransferase
901253	Spy1137		-15.4		-7.5	Purine permease
901254	Spy1138				-3.1	Thiamine biosynthesis lipoprotein
901255	Spy1139	-3.5			-3.6	4-oxalocrotonate tautomerase
901256	Spy1140				-3.3	Thymidine kinase
901264	Spy1148		-3.5			ABC transporter ATP-binding protein
901265	Spy1149		-3.5			ABC transporter ATP-binding protein
901267	Spy1151				-3.4	L-lactate dehydrogenase
901271	Spy1156				-4.2	Hypothetical protein
901272	Spy1157				-4.6	Signal peptide protein
901273	Spy1158	-3.0				DeoR family transcriptional regulator
901274	Spy1159	-4.9		-5.7		Hemolysin HylIII
901275	Spy1160	-4.9		-5.7		Cytoplasmic protein
901278	Spy1163				-15.2	Smf family DNA processing protein
901280	Spy1168				-6.2	Putative transcriptional regulator
901282	Spy1170			-5.2		D-lactate dehydrogenase
901287	Spy1175				-3.7	Membrane protein
901288	Spy1176				-3.7	Methylmalonyl-CoA decarboxylase, gamma-subunit
901292	Spy1180			-3.6		Mg2+/citrate complex transporter
901293	Spy1181	-5.6		-21.0		Hypothetical protein
901294	Spy1183	-7.0		-6.2		Acetyl-CoA carboxylase biotin carboxyl carrier protein subunit
901295	Spy1184			-3.3	-5.5	Decarboxylase
901302	Spy1193				-3.0	Hypothetical protein
901303	Spy1196				-7.1	Site-specific tyrosine recombinase XerC
901306	Spy1201				-4.0	DNA-binding protein ylxM
901310	Spy1205				-3.1	UDP-N- acetylmuramoyl-pentapeptide-lysine N(6)-alanyltransferase
901316	Spy1212		-3.7			Cardiolipin synthetase
901317	Spy1213		-5.1			Formate--tetrahydrofolate ligase
901318	Spy1214		-3.2			Lipoate-protein ligase
901327	Spy1223				-3.2	Membrane protein
901329	Spy1225		-4.9			Sugar ABC transporter permease

901335	Spy1233	-3.6				Pantothenate kinase
901336	Spy1234				-4.6	30S ribosomal protein S20
901340	Spy1240		-3.1			Phosphate uptake regulatory protein PhoU
901341	Spy1241		-3.2			Phosphate transporter ATP-binding protein PstB
901342	Spy1242		-3.5			Phosphate transporter ATP-binding protein PstB2
901355	Spy1255				-3.8	Peptide ABC transporter permease
901356	Spy1257	-3.0		-3.8	-4.6	ABC transporter ATP-binding protein
901357	Spy1258	-5.6	3.7	4.8	-3.1	TetR/AcrR family transcriptional regulator
901358	SPy1259				-3.1	Transcriptional regulator
901364	Spy1265	-3.3				Transglycosylase associated protein
901366	Spy1270		-9.1		-7.4	Na(+)-linked D-alanine glycine permease
901369	Spy1274		-3.4			Amino acid ABC transporter substrate-binding protein
901370	Spy1275		-3.5			Amino acid ABC transporter ATP-binding protein
901385	Spy1292	-3.7				4-alpha-glucanotransferase MalM
901386	Spy1293	-3.9				Maltose operon transcriptional repressor MalR
901391	Spy1298				-3.0	Maltodextrose utilization protein MalA
901396	Spy1306	-3.5		-4.0	-3.7	Maltose/maltodextrin-binding protein MalX
901405	Spy1319			-5.9	-4.4	
901410	Spy1326			-3.4		Outer surface protein
901411	Spy1328		5.5			Beta-glucosidase BglA.2
901413	Spy1332				-3.3	UDP-N-acetylglucosamine 1-carboxyvinyltransferase
901415	Spy1335			-9.1		Conserved hypothetical protein - transposon IS861 associated
901419	Spy1339	-9.0		-38.3		Diaminopimelate epimerase
901432	Spy1357	-9.0				Protein G-like alpha 2M-binding protein (GRAB)
901441	Spy1367				-4.6	Membrane protein
901442	Spy1368				-3.2	Uridine kinase
901448	Spy1374				-3.7	Glutaredoxin NrdH
901449	Spy1375				-3.1	Ribonucleotide-diphosphate reductase subunit alpha NrdE.2
901450	Spy1378		-3.1		-4.6	Ribonucleotide-diphosphate reductase subunit beta
901451	Spy1379		-3.1		-4.6	

901459	Spy1392		-27.7		-7.8	Oxalate/formate antiporter
901461	Spy1395				-3.3	Transcriptional factor
901463	Spy1399				-3.8	N-acetylglucosamine-6-phosphate isomerase
901466	Spy1402	-3.2				Hypothetical protein
901467	Spy1404	-3.2				Hypothetical protein
901468	Spy1405	-3.2				Hypothetical protein
901469	Spy1406	-3.2				Superoxide dismutase (Fe/Mn) SodA
901470	Spy1407	-3.3				DNA polymerase III subunit delta
901471	Spy1408		-9.8			Competence protein ComEC
901472	Spy1409		-9.8			Competence protein ComEA
901479	Spy1419				-3.8	Integral membrane family protein
901482	Spy1422				-3.2	Recombination protein RecR
901483	Spy1423				-3.4	Similar to penicillin binding proteins
901486	Spy1427		3.2			Transcriptional regulator
901489	Spy1434				-3.1	Heavy metal-transporting ATPase
901491	Spy1437				-3.2	Membrane protein (prophage 370.3)
901496	Spy1443			-3.6	-3.6	Hypothetical protein (prophage 370.3)
901498	Spy1445				-4.2	Phage associated hyaluronidase
901499	Spy1446				-4.2	Phage minor structural protein, N-terminal domain protein (prophage 370.3)
901500	Spy1447				-4.2	Hypothetical protein (prophage 370.3)
901501	Spy1448				-4.2	Hypothetical protein (prophage 370.3)
901502	Spy1449				-4.2	Hypothetical protein (prophage 370.3)
901512	Spy1460		3.1			Terminase (prophage 370.3)
901520	Spy1469			-4.6		Hypothetical protein (prophage 370.3)
901522	Spy1471	3.6				Membrane protein (prophage 370.3)
901530	Spy1481			-10.3	-4.0	Hypothetical protein (prophage 370.3)
901531	Spy1482			-10.3	-4.0	DNA replication protein DnaD (prophage 370.3)
901539	Spy1489				-3.1	Histone-like DNA-binding protein
901545	Spy1496	-3.2				Repressor protein
901546	Spy1497	-3.2				Hemolysin HlyA1
901547	Spy1498	-3.2				Geranyltranstransferase
901548	Spy1499	-3.2				Exodeoxyribonuclease VII small subunit
901549	Spy1500	-3.2				Exodeoxyribonuclease VII large subunit
901551	Spy1503	-4.0				Phosphomannomutase
901555	Spy1508				-3.5	Hypothetical cytosolic protein

901570	Spy1526				-4.1	Membrane protein
901576	Spy1532		-3.5			Prepilin peptidase
901579	Spy1535		-3.6			Ribose transport operon repressor
901583	Spy1539		-3.7			Asparagine synthetase A (aspartate--ammonia ligase) AsnA
901693	Spy1543	-1.8*	4.1			Arginine/ornithine antiporter ArcD
901696	Spy1544	-1.7*		-3.1		Ornithine carbamoyltransferase ArcB
901712	Spy1546	-26.1		-5.0		Acetyltransferase
901715	Spy1547	-10.1		-4.9		Arginine deiminase ArcA
901748	Spy1551	-3.9				tRNA synthetase subunit beta
901782	Spy1558				-8.6	Thiol:disulfide interchange protein TlpA
901827	Spy1580				-3.7	Acetate kinase
901839	Spy1591				-4.8	ABC transporter substrate-binding protein
901841	Spy1592				-4.8	ABC transporter substrate-binding protein
901842	Spy1593				-5.0	Sugar-binding transport protein
901843	Spy1595				-2.8*	Sugar-binding transport protein
901844	Spy1596				-7.9	Transcriptional regulator
901858	Spy1607	-4.4			-1.6*	Recombination regulator RecX
901859	Spy1608	-1.2*			-4.4	Hypothetical protein
901860	Spy1610			4.0		Transposase
901861	Spy1613	-6.8		-4.0		Sigma 54 modulation protein /Ribosome-associated factor Y
901862	Spy1615		-4.8			Late competence protein ComFC
901863	Spy1616		-4.8			DNA uptake late competence protein ComFA
901866	Spy1618				-4.8	O-acetylserine lyase
901873	Spy1623				-3.7	Transporter YvqF
901898	Spy1644		-4.8			Methyltransferase
901908	Spy1656		-4.3			Membrane protein
901909	Spy1657		-2.0*		-3.1	Amino acid ABC transporter ATP-binding protein
901910	Spy1658		-2.0*		-3.1	Amino acid ABC transporter permease
901912	Spy1662		-3.1			Phospho-N-acetylmuramoyl pentapeptide transferase MraY
901922	Spy1675	-3.2				Enterocin A immunity protein
901925	Spy1678	-3.3				Transaldolase
901926	Spy1680	-3.0		-4.8		Trans-acting positive regulator
901927	Spy1681	-3.0		-4.8		NADH peroxidase/NADH oxidase
901928	Spy1682		-4.5			Glycerol uptake facilitator GlpF
901929	Spy1683				-3.4	Alpha-glycerophosphate oxidase GlpO

901930	Spy1684				-3.6	Glycerol kinase
901946	Spy1707				-4.3	Galactose-6-phosphate isomerase subunit LacB
901947	Spy1708				-4.0	Galactose-6-phosphate isomerase subunit LacA
901948	Spy1709			-3.2		PTS system galactose-specific transporter subunit IIC
901949	Spy1710	-4.1				PTS system galactose-specific transporter subunit IIB
901950	Spy1711	-4.1				PTS system galactose-specific transporter subunit IIA
901952	Spy1714				-3.5	Copper chaperone
901961	Spy1725				-4.0	Ribosome maturation protein RimP
901964	Spy1728		-3.2			ABC transporter permease
901968	Spy1733		-5.0			Transcriptional regulator
901969	Spy1734		-3.7			Acetyltransferase
901970	Spy1735		-3.7			ATP/GTP hydrolase
901971	Spy1736		-32.9		-11.1	Guanine-hypoxanthine permease PbuG
901975	Spy1740				-3.2	PTS system mannose-specific transporter subunit IID
901977	Spy1742	-3.4				Seryl tRNA synthetase SerS
901978	Spy1743	-3.8	-3.9	-3.0	-3.5	Acetyl-coenzyme A carboxylase carboxyl transferase subunit alpha AccA
901979	Spy1744	-3.8	-3.9	-3.0	-3.5	Acetyl-coenzyme A carboxylase carboxyl transferase subunit beta AccD
901980	Spy1745	-3.6	-4.3	-3.0		Biotin carboxylase (A subunit of acetyl-CoA carboxylase) AccC
901981	Spy1746	-3.2	-3.8	-3.3	-4.2	(3R)-hydroxymyristoyl-(acyl carrier protein) dehydratase FabZ
901982	Spy1747	-3.2	-3.8	-3.3	-4.2	biotin carboxyl carrier protein of acetyl-CoA carboxylase AccB
901983	Spy1748		-4.1			3-oxoacyl-ACP synthase FabF
901984	Spy1749		-4.0			3-ketoacyl-ACP reductase FabG
901986	Spy1750		-4.0			Malonyl CoA-ACYL carrier protein transacylase FabD
901987	Spy1751		-4.7			Trans-2-enoyl-ACP reductase II FabK
901988	Spy1753				-3.2	Acyl carrier protein AcpP
901989	Spy1754		-3.4			3-oxoacyl-ACP synthase FabH
901990	Spy1755		-3.0			MarR family transcriptional regulator
901992	Spy1759				-3.4	Molecular chaperone DnaJ
902008	Spy1776		10.7		-3.9	Pyrazinamidase/nicotinamidase
902011	Spy1780	-3.2				Universal stress protein UspA

902016	Spy1785		-3.6			ATP-dependent DNA helicase RecG
902028	Spy1802		-3.3		-3.4	Alanine racemase
902029	Spy1804		-3.3		-3.4	Holo-[acyl-carrier protein] synthase AcpS
902034	Spy1811			-3.5	-5.0	Fructokinase
902035	Spy1813			-3.9	-4.0	Endo-beta-N-acetylglucosaminidase F2
902036	Spy1815			-3.7	-5.1	PTS system sucrose-specific transporter subunit IIABC
902047	Spy1829				-3.7	30S ribosomal protein S18
902050	Spy1832				-6.6	Hypothetical protein
902057	Spy1840				-3.8	Seryl-tRNA synthetase
902066	Spy1852		-5.2			Sulfite exporter TauE/SafE domain protein
902067	Spy1854				-3.7	Glycerol uptake facilitator protein
902068	Spy1856	-18.5	3.1	-6.8		Multidrug resistance protein NorA homolog
902070	Spy1858		-3.2			XAA-Pro dipeptidyl-peptidase PepXP
902078	Spy1868				-3.0	Nucleoside transporter
902079	Spy1869		-3.4			Uridine phosphorylase
902085	Spy1875		-3.5		-3.6	Hypersensitive-induced response proteins
902086	Spy1876		-3.4		-2.3*	Zn-dependent hydrolase
902092	Spy1884				-3.0	Hypersensitive-induced response proteins
902095	Spy1888				-5.2	50S ribosomal protein L28
901595	Spy1911				-3.3	ABC transporter permease SalY
901596	Spy1912				-3.3	ABC transporter ATP-binding protein SalX
901597	Spy1913				-3.3	ABC transporter, ATP-binding protein
901599	Spy1915				-6.6	Lantibiotic SalA
901600	Spy1916				-4.7	6-phospho-beta-galactosidase LacG
901604	Spy1921				-6.0	Tagatose-6-phosphate kinase LacC.2
901606	Spy1922				-3.0	Galactose-6-phosphate isomerase subunit LacB
901607	Spy1923	-3.3			-5.4	LacA subunit
901622	Spy1942		-4.4		-5.1	Hypothetical protein
901627	Spy1949				-4.3	PTS system ascorbate-specific transporter subunit IIC
901628	Spy1950				-3.9	PTS system transporter subunit IIB
901629	Spy1952				-3.9	PTS fructose transporter subunit IIA
901643	Spy1971	-3.8				Bacteriocin transporter
901646	Spy1976	-3.1				Multiple sugar-binding transport ATP-binding protein MsmK
901651	Spy1983	4.9				Collagen-like surface protein SclA
901653	Spy1985			-3.2		Exodeoxyribonuclease III

901657	Spy1989				-3.1	Hypothetical protein
901661	Spy1994				-3.3	Repressor protein Pai1
901677	Spy2018	11.1	11.4	12.2	5.0	M-protein serotype 1 (*emm1*)
901689	Spy2038		-3.3		57.6	Spi SpeB protease inhibitor
901690	Spy2039				143.9	Pyrogenic exotoxin B
901691	Spy2040				135.9	Hypothetical protein
901692	Spy2041	-5.6			16.6	Hypothetical protein
901694	Spy2042	-3.6				Transcription regulator RopB/Rgg1
901700	Spy2049			-3.1		Pyruvate formate-lyase 2
901701	Spy2050			-5.0		PTS system cellobiose-specific transporter subunit IIC
901706	Spy2055			-3.2		Pyruvate formate-lyase activating protein
901709	Spy2060	-3.5				Endoribonuclease L-PSP
901719	Spy2077				-5.5	Cold shock protein
901720	Spy2079	-3.4				Alkyl hydroperoxidase AhpC
901722	Spy2081		4.7			Imidazolone-5-propionate hydrolase HutI
901723	Spy2082			-22.8	-4.4	Urocanate hydratase HutU
901724	Spy2083	4.3		-11.8		Glutamate formiminotransferase
901725	Spy2084			-10.9		Formiminotetrahydrofolate cyclodeaminase
901726	Spy2085	-3.2	-4.9			Formate-tetrahydrofolate ligase Fhs.2
901728	Spy2088			-7.7	-6.6	Cationic amino acid transporter protein
901729	Spy2089	-3.7		-12.5		Histidine ammonia-lyase HutH
901731	Spy2091				-3.3	Regulatory protein
901734	Spy2093				-3.0	Elongation factor Tsf
901736	Spy2096		-3.8			Trehalose-6-phosphate hydrolase
901737	Spy2097	-3.4		-7.2		PTS system transporter subunit II
901739	Spy2102		-5.7		-3.0	MarR family transcriptional regulator
901740	Spy2103	-4.5			-3.4	Glyoxalase
901742	Spy2105		-9.0		-3.0	Anaerobic ribonucleotide reductase activator protein NrdG
901743	Spy2106		-9.0		-3.4	GNAT family acetyltransferase
901745	Spy2107		-8.7		-3.5	Oxidoreductase
901746	Spy2110				-3.2	Anaerobic ribonucleoside triphosphate reductase
901757	Spy2118		-3.7		-3.5	DNA-3-methyladenine glycosidase I Tag
901758	Spy2119		-4.3		-1.5*	Holiday junction DNA helicase RuvA
901759	Spy2120		-5.6		-4.2	Major facilitator family protein LmrP
901760	Spy2121		-3.1		-2.6*	DNA mismatch repair protein MutL
901762	Spy2122	107.9	40.7	75.8	111.9	SpyCIM1 integrase

901763	Spy2125	68.2	81.5	87.8	95.3	SpyCIM1 repressor protein cI
901764	Spy2126		12.2	16.5	48.4	SpyCIM1 antirepressor protein
901765	Spy2127	39.8	34.1	28.6	56.0	SpyCIM1 protein
901766	Spy2128	32.7	34.1	31.8	65.0	SpyCIM1 protein
901768	Spy2129	32.7	34.1	31.8	65.0	SpyCIM1 protein
901769	Spy2130	14.2		23.1	31.8	SpyCIM1 protein
901770	Spy2131	14.2		23.1	31.8	SpyCIM1 protein
901771	Spy2132			11.0	28.7	SpyCIM1 protein
901772	Spy2133				24.2	SpyCIM1 protein
901773	Spy2134			16.5	30.3	SpyCIM1 protein
901774	Spy2135	83.8	41.7	106.5	216.3	SpyCIM1 replication protein
901775	Spy2136	83.8	41.7	106.5	216.3	SpyCIM1 DNA primase
901776	Spy2140		49.9	19.8	49.9	SpyCIM1 protein
901777	Spy2142		19.7		19.7	SpyCIM1 protein
901778	Spy2144	18.5	15.9	11.0	21.2	SpyCIM1 protein
901779	Spy2145	18.5	15.9	11.0	21.2	SpyCIM1 HTH motif protein
901780	Spy2147	59.6	57.6	85.6	86.2	SpyCIM1 protein
901787	Spy2153		-3.4			Membrane protein
901797	Spy2164		-4.4			Hypothetical protein
901805	Spy2176	-4.4	-3.3	-5.3	-6.5	YhgE/Pip domain-containing protein
901806	Spy2177				-3.1	TetR/AcrR family transcriptional regulator
901831	Spy2185		-4.4		-4.3	Glucose inhibited division protein A GidA
901835	Spy2186		-3.4		-1.7*	MutT/nudix family protein
901848	Spy2190				-3.2	L-serine dehydratase subunit alpha
901851	Spy2191		-4.9		-5.1	Transglycosylase
901875	Spy2200	4.6		2.3*	-10.6	Capsule biosynthesis HasA
901876	Spy2201	6.2		4.9	-2.8*	Capsule biosynthesis Has*B*
901878	Spy2202	6.0		2.7*	-3.2	Capsule biosynthesis HasC
901894	Spy2205		-3.3			Glucose uptake family protein
901899	Spy2207		-3.5		-3.1	Tryptophanyl-tRNA synthetase
901985	Spy2214		-4.3			Transposase domain

[1] GenBank identifier
[2] Gene locus tag based upon the SF370 annotated genome
[3] Ratio EL – SF370SmR/CEM1Δ4 when $A_{600\ nm} = 0.2$; Ratio LL - SF370SmR/CEM1Δ4 when $A_{600\ nm} = 0.5$
[4] Not detected
* Included as part of polycistronic mRNA even though less than 3-fold difference between SF370SmR and CEM1Δ4
