# Supplementary material for: Elimination of Chromosomal Island SpyCIM1 from Streptococcus pyogenes Strain SF370 Reverses the Mutator Phenotype and Alters Global Transcription
Source: PLoS One. 2015 Dec 23;10(12):e0145884. doi: 10.1371/journal.pone.0145884 (PMC4689407; doi:10.1371/journal.pone.0145884)
Supplement: S3 Table — The analysis was done using GeneSifter as above. (PDF) [file pone.0145884.s007.pdf]

S3 Table.

KEGG Pathway	No. of genes	Down	Up	Gene Set	z-score (Down)	z-score (Up)
Metabolic pathways	33	29	4	288	3.6	1.07
Microbial metabolism in diverse environments	14	13	1	70	4.5	0.45
Phosphotransferase system (PTS)	8	8	0	33	4.38	-0.57
Amino sugar and nucleotide sugar metabolism	7	5	2	28	2.59	3.48
Propanoate metabolism	6	5	1	15	4.38	2.33
Fatty acid biosynthesis	5	5	0	11	5.41	-0.33
Fructose and mannose metabolism	5	5	0	15	4.38	-0.38
Pyruvate metabolism	5	5	0	23	3.12	-0.48
Starch and sucrose metabolism	4	2	2	15	1.14	5.03
Aminobenzoate degradation	3	2	1	10	1.81	2.99
Ascorbate and aldarate metabolism	3	2	1	9	1.99	3.18
Mismatch repair	3	3	0	16	2.09	-0.39
Benzoate degradation	2	2	0	6	2.75	-0.24
Biotin metabolism	2	2	0	6	2.75	-0.24
Histidine metabolism	2	1	1	6	1.06	4.01
One carbon pool by folate	2	1	1	10	0.49	2.99
Pentose and glucuronate interconversions	2	0	2	10	-0.82	6.29
Tyrosine metabolism	2	2	0	7	2.45	-0.26
Butanoate metabolism	1	0	1	13	-0.94	2.55
Chloroalkane and chloroalkene degradation	1	1	0	2	2.56	-0.14
Naphthalene degradation	1	1	0	2	2.56	-0.14
Riboflavin metabolism	1	1	0	2	2.56	-0.14
Taurine and hypotaurine metabolism	1	1	0	2	2.56	-0.14
