# Supplementary material for: Elimination of Chromosomal Island SpyCIM1 from Streptococcus pyogenes Strain SF370 Reverses the Mutator Phenotype and Alters Global Transcription
Source: PLoS One. 2015 Dec 23;10(12):e0145884. doi: 10.1371/journal.pone.0145884 (PMC4689407; doi:10.1371/journal.pone.0145884)
Supplement: S4 Table — The analysis was done using GeneSifter as above. (PDF) [file pone.0145884.s008.pdf]

S4 Table.

KEGG Pathway	No. of genes	Down	Up	Gene Set	z-score (Down)	z-score (Up)
Metabolic pathways	20	18	2	288	2.53	-2.17
Aminoacyl-tRNA biosynthesis	11	0	11	86	-2.05	7.59
Microbial metabolism in diverse environments	7	7	0	70	2.66	-1.28
Phosphotransferase system (PTS)	6	6	0	33	4.21	-0.85
Propanoate metabolism	6	6	0	15	7.12	-0.56
Pyruvate metabolism	5	5	0	23	4.37	-0.7
Fatty acid biosynthesis	4	4	0	11	5.46	-0.48
Starch and sucrose metabolism	4	3	1	15	3.16	1.28
Aminobenzoate degradation	3	3	0	10	4.18	-0.46
Butanoate metabolism	3	3	0	13	3.5	-0.52
Fructose and mannose metabolism	3	3	0	15	3.16	-0.56
Histidine metabolism	3	3	0	6	5.72	-0.35
Arginine and proline metabolism	2	2	0	11	2.39	-0.48
One carbon pool by folate	2	2	0	10	2.57	-0.46
Tyrosine metabolism	2	2	0	7	3.3	-0.38
Chloroalkane and chloroalkene degradation	1	1	0	2	3.29	-0.2
Fatty acid metabolism	1	1	0	4	2.12	-0.29
Naphthalene degradation	1	1	0	2	3.29	-0.2
