# Supplementary material for: Elimination of Chromosomal Island SpyCIM1 from Streptococcus pyogenes Strain SF370 Reverses the Mutator Phenotype and Alters Global Transcription
Source: PLoS One. 2015 Dec 23;10(12):e0145884. doi: 10.1371/journal.pone.0145884 (PMC4689407; doi:10.1371/journal.pone.0145884)
Supplement: S5 Table — The analysis was done using GeneSifter as above. (PDF) [file pone.0145884.s009.pdf]

S5 Table.

KEGG Pathway	No. of genes	Down	Up	Gene Set	z-score (Down)	z-score (Up)
Aminoacyl-tRNA biosynthesis	34	34	0	86	7.61	-0.68
Purine metabolism	14	14	0	49	3.22	-0.5
Fatty acid biosynthesis	10	10	0	11	7.59	-0.23
Propanoate metabolism	5	4	1	15	1.52	3.55
Ribosome	4	4	0	71	-2.05	-0.61
Biotin metabolism	3	3	0	6	2.63	-0.17
Glycolysis / Gluconeogenesis	2	1	1	24	-1.36	2.7
Aminobenzoate degradation	1	0	1	10	-1.26	4.44
Butanoate metabolism	1	0	1	13	-1.44	3.85
Histidine metabolism	1	0	1	6	-0.97	5.83
Two-component system	1	0	1	30	-2.21	2.35
