# Supplementary material for: Elimination of Chromosomal Island SpyCIM1 from Streptococcus pyogenes Strain SF370 Reverses the Mutator Phenotype and Alters Global Transcription
Source: PLoS One. 2015 Dec 23;10(12):e0145884. doi: 10.1371/journal.pone.0145884 (PMC4689407; doi:10.1371/journal.pone.0145884)
Supplement: S6 Table — The analysis was done using GeneSifter as above. (PDF) [file pone.0145884.s010.pdf]

S6 Table.

KEGG Pathway	No. of genes	Down	Up	Gene Set	z-score (Down)	z-score (Up)
Ribosome	17	17	0	71	2.18	-0.5
Purine metabolism	16	16	0	49	3.54	-0.41
Aminoacyl-tRNA biosynthesis	12	10	2	86	-0.99	3.59
Ascorbate and aldarate metabolism	5	5	0	9	3.4	-0.17
