# Supplementary material for: Elimination of Chromosomal Island SpyCIM1 from Streptococcus pyogenes Strain SF370 Reverses the Mutator Phenotype and Alters Global Transcription
Source: PLoS One. 2015 Dec 23;10(12):e0145884. doi: 10.1371/journal.pone.0145884 (PMC4689407; doi:10.1371/journal.pone.0145884)
Supplement: S7 Table — The cDNA preparations used for RNA-seq analysis were analyzed by qRT-PCR for comparing the expression of nga, slo, norA, emm1, speB, and hasB in SF370SmR to CEM1Δ4 as described in the Methods. (PDF) [file pone.0145884.s011.pdf]

S7 Table.

	Gene	qRT-PCR fold change (SF370SmR/CEM1Δ4)	RNA-seq fold change (SF370SmR/CEM1Δ4)*
**37 °C Early Log**			
	nga	2.0	2.6
	slo	4.5	3
	norA	-14.3	-18.5
	emm	11.9	11.1
	speB	**	**
	hasB	2.1	6.2
**37 °C Late Log**			
	nga	-1.9	1.5
	slo	-2.7	1.2
	norA	3.5	3.1
	emm	36.3	11.4
	speB	1.0	1
	hasB	-1.6	1
**39 °C Early Log**			
	nga	3.1	2.3
	slo	7.9	3.6
	norA	-3.8	-6.8
	emm	27	12.2
	speB	**	**
	hasB	1.7	4.9
**39 °C Late Log**			
	nga	1.9	1.4
	slo	1.0	1.3
	norA	-1.6	-2.8
	emm	79	5
	speB	251	143.9
	hasB	-4.4	-2.8

* Values calculated by Gene Sifter as described in Methods. The identical cDNA preparations were used for qRT-PCR analysis and RNA-seq analysis

** Not expressed in EL
