# Supplementary material for: Elimination of Chromosomal Island SpyCIM1 from Streptococcus pyogenes Strain SF370 Reverses the Mutator Phenotype and Alters Global Transcription
Source: PLoS One. 2015 Dec 23;10(12):e0145884. doi: 10.1371/journal.pone.0145884 (PMC4689407; doi:10.1371/journal.pone.0145884)
Supplement: S8 Table — Three independent cultures of SF370SmR and of CEM1Δ4 were grown at 39°C; samples were harvested for RNA isolation when the culture density (A600 nm) was 0.2 (EL) and again when the density was 0.5 (LL). An addition sample was harvested one-hour post LL (Stationary). After conversion of the RNA to cDNA, qRT-PCR was used to compare expression levels of the listed genes between SF370SmR and CEM1Δ4. Values are the average and standard deviation of the fold-difference between the two stains. (PDF) [file pone.0145884.s012.pdf]

S8 Table.

Gene	EL	LL	Stationary
nga	2.7 ± 1.1*	1.3 ± 2.4	2.3 ± 1.6
slo	2.6 ± 0.7	1.3 ± 1.9	1.6 ± 0.2
norA	-31.1 ± 8.3	-0.6 ± 1.6	-2.7 ± 0.4
emm	11.4 ± 5.9	22.5 ± 7.5	5.0 ± 3.4
speB	**	18.3 ± 11.9	-2.2 ± 0.4
hasB	2.7 ± 1.1	0.4 ± 1.1	-2.2 ± 2.9

EL – Early logarithmic growth; A_{600} = 0.2 nm
LL – Late logarithmic growth; A_{600} = 0.5 nm
Stationary – One hour after LL sample
* qRT-PCR fold change (SF370SmR/CEM1Δ4)
** Not expressed in EL [46-48]
